# Native Capillary Nanogel Electrophoresis Assay of Inhibitors of Neuraminidases Derived from H1N1 and H5N1 Influenza A Pandemics

**DOI:** 10.1021/acs.analchem.4c06127

**Published:** 2025-02-28

**Authors:** Laura N. Taylor, Lisa A. Holland, Makenzie T. Witzel

**Affiliations:** C. Eugene Bennett Department of Chemistry, West Virginia University, Morgantown, West Virginia 26505, United States

## Abstract

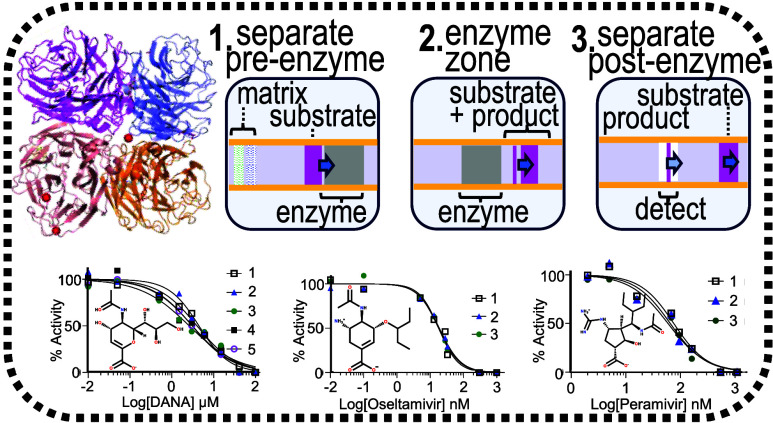

Tetrameric neuraminidases cleave the end-capping sialylated monomer from oligosaccharide ligands at the surface of a host cell infected by the influenza A virus. This cleavage releases the replicated virions from the host cell, making drugs that inhibit neuraminidase function effective to treat influenza A infections. A capillary electrophoresis separation-based assay is reported that maintains the native structure of tetrameric viral neuraminidases derived from H1N1 or H5N1 influenza A pandemics which convert, in-real time, a substrate that mimics 6′-sialyllated threonine-linked glycans on human cells. The assay integrates the enzyme reaction with the separation and is operated using a background electrolyte containing 100 mM NaCl with a thermally reversible nanogel in a 10 μm inner diameter fused silica capillary. In addition to defining the 0.4 nL reaction zone maintained at 37 °C, the nanogel medium resolves the substrate from contaminants as well as the substrate from the product before and after the enzymatic conversion. The enzyme activity is quantifiable based on the percent conversion observed in the presence of a range of inhibitor concentrations. For 1918 H1N1 (A/Brevig Mission/1/18) neuraminidase, the inhibition constant of the transition state analog 2,3-dehydro-2-deoxy-*N*-acetylneuraminic acid (DANA) is 3.5 ± 0.8 μM (*n* = 5). The inhibition constants for oseltamivir acid (inhibiting compound of Tamiflu) and peramivir (Rapivab) are 18.2 ± 0.5 nM (*n* = 3) and 67 ± 8 nM (*n* = 3), respectively. For 2004 H5N1 (A/Vietnam/1203/2004) neuraminidase, which contained a foreign tetramerization domain to maintain the structure, the inhibition constant for peramivir is 5.4 nM.

Protein complexes are prevalent in physiological systems,^1^ making them attractive drug targets. Multimeric protein structures are particularly important to influenza A infections as they interact with sialylated ligands to bind to the host cell.^2^ Viral neuraminidase, which is an enzyme assembled as a tetrameric protein structure,^3,4^ is responsible for the release of newly produced virions at the host cell surface through the enzymatic cleavage of sialylated ligands at the cell surface. The health effects of influenza A infections vary widely. Since the broadly publicized occurrence of influenza A in 1918, pandemics of varying strains and subtypes have materialized across the globe. As a result, inhibitors of viral neuraminidase are developed to treat infections, and these drugs are stockpiled^5^ as a precautionary measure to contain outbreaks. Some mutations of influenza A cause resistance to current inhibitors and efforts to develop additional inhibitors are ongoing.^5^

Bioanalytical assays can be used to determine the efficacy of current neuraminidase inhibitors against emerging viruses collected during the influenza A season as well as archived viruses attributed to prior influenza A pandemics. Neuraminidase inhibition assays commonly employ intact viruses.^6^ However, assays with intact viruses must be performed under stringent biosafety guidelines to prevent exposure. Analyzing neuraminidase rather than intact virus, mitigates this risk, and is essential for the study of pandemic viruses. Recombinant technologies are vital to studies of influenza A, shedding light on tetrameric stability^7^ and the virulence associated with glycan-stabilization of tetrameric neuramindase.^8^ Advancing biotechnology tools, such as recombinant proteins, require new bioanalytical methods to address the challenges and the increased demand to mitigate viral pandemics.^9^

Capillary electrophoresis is a bioanalytical tool that can quantify the activity of a wide range of enzymes, substrates, and inhibitors using a separation-based assay to resolve the product and substrate following the enzyme conversion.^10^ Several advantages are realized with capillary electrophoresis enzyme assays. This automated and miniaturized separation method is suitable to resolve and subsequently quantify the product from the unreacted substrate generated from an enzyme reaction. The method typically has a total separation volume less than 0.5 μL, and when the enzyme reaction is integrated into the separation capillary the conversion in an enzyme zone of only a few nanoliters can occur in real time. With the increased availability of multimeric and recombinant enzymes microscale electrophoresis assays must be adapted to preserve native structures, especially neuraminidase which is a tetrameric protein complex. This requires the assay to be conducted in high salt solutions that include enzyme activators (e.g., calcium chloride), a specific pH and temperature. Traditionally, capillary electrophoresis separations are limited to low ionic strength background electrolytes, keeping the separation current low in order to avoid Joule heating that occurs in the presence of the high electric fields that efficiently drive the separation. Additionally, glycosylated substrate molecules are typically separated by capillary electrophoresis using viscous gels. While the separation current decreases with the inner diameter of the separation channel, narrow bore capillaries limit the use of viscous gels which are frequently required to separate glycosylated substrate molecules. This results from the high back pressure, which scales with the square of the capillary radius, that is used to fill the capillary.

The purpose of the bioanalytical approach outlined in this report is to develop enabling technology that emulates the physiological conditions relevant to viral neuraminidase tetrameric enzyme without requiring extensive biological containment. By designing the assay in a capillary nanogel system, the effectiveness of drugs to inhibit neuraminidase associated with either an archived or an emerging influenza virus can be quantified. Several innovations are outlined in this report to achieve this purpose. For the first time, the efficacy of therapeutics against pandemic H1N1 and H5N1 influenza A is measured in real-time within a subnanoliter reaction zone of recombinant neuraminidase without the need for the stringent biological containment measures that are required when evaluating neuraminidases in intact viruses. This achievement is possible with a novel electrophoresis-based assay specifically designed to maintain the enzyme reaction under native conditions that mimic the enzyme cleavage at the host cell surface. A self-assembled phospholipid nanogel compatible with a high salt (100 mM NaCl) nanogel was developed for in-capillary enzyme reactions. The nanogel also provides a microscale separation to resolve and quantify the amount of product and substrate remaining after the enzyme reaction. The thermally reversible viscosity of this high salt nanogel is integrated within a 10 μm inner diameter separation capillary, preventing Joule heating while also enabling a practical method to introduce the viscous gel into the narrow bore capillary.

This report outlines the approach to create a discrete reaction zone in highly viscous nanogel preparations with a commercial capillary electrophoresis instrument. The inhibitor is introduced separately from the enzyme and mixed in the capillary, making it possible to use a single enzyme preparation to quantify the reduced activity observed at multiple concentrations of a particular inhibitor. The method is applied to recombinant enzymes derived from the 1918 H1N1 as well as the 2004 H5N1 influenza A pandemics. The utility of these recombinant enzymes as substitutes for intact virus assays is demonstrated with the active ingredients of modern therapeutics, generating nanomolar inhibition constants, some of which were not available at the time of each pandemic.

## Materials and Methods

### Reagents and Materials

The 6′-sialyllactose sodium salt (OS04398) and oseltamivir acid (FO26594 lot 265941802, purity 98.9%) were purchased from Carbosynth (Biosynth Carbosynth Ltd., San Diego, CA). TRIS hydrochloride (410304) and sodium chloride (S271–500) were purchased from Fisher Scientific (Thermo Fisher Scientific, Pittsburgh, PA). Recombinant influenza A virus neuraminidase proteins H1N1 (4858-NM-005 lot RJJ0822031) and H5N1 (7597-NM-010 lot DCLS0121052) were purchased from R&D Systems (Minneapolis, MN). The DANA (D9050), peramivir (SML2486 batch 0000135861 purity 99.8%), acetic acid (A6283), 8-aminopyrene-1,3,6-trisulfonic acid trisodium salt (APTS, A7222), 2-(N-morpholino)ethanesulfonic acid (MES, M8902), triethylamine (471283), acetonitrile (34851), methanol (646377) and sodium cyanoborohydride (156159) were purchased from Sigma-Aldrich (St. Louis, MO). Sodium acetate, anhydrous (7510-OP) was obtained from EM Sciences (Millipore Sigma, Burlington, MA). Phospholipids 1,2-dihexanoyl-*sn*-glycero-3-phosphocholine (DHPC, 850305P) and 1,2-dimyristoyl-*sn*-glycero-3-phosphocholine (DMPC, 850345P) were purchased from Avanti Polar Lipids (Alabaster, AL). All solutions were dissolved in purified deionized water (18 MΩ/cm) obtained from an Elga Purelab and Veolia Chorus water system (Lowell, MA). The 6′-sialyllactose substrate was derivatized with APTS and purified as previously reported.^11^ The pH of TRIS and MES buffers was adjusted at ambient temperature, typically from 20 to 22 °C, using a standard pH meter and 3-point calibration.

### Nanogel and Enzyme Preparations

Phospholipids were used to prepare nanogels similar to a previously reported method.^12,13^ These phospholipid nanogels were made at molar ratios, *q* = DHPC/DMPC, of *q* = 0.5 and *q* = 2.5. The *q* = 0.5 phospholipid preparation, which was used to passivate the separation capillary, was diluted to a final lipid concentration of 5% mass/volume using 50 mM sodium acetate buffered to pH 5 and stored at −20 °C in 100 μL aliquots for up to 2 weeks. Prior to use, calcium chloride was added to the 5% phospholipid to bring the final concentration to 1.25 mM as it is a fusogenic agent used for semipermanent phospholipid coatings. The *q* = 2.5 preparation, which was used as the separation medium, was diluted and stored at −20 °C in 50 μL aliquots for up to 2 weeks. Calcium chloride was included in the nanogel because it was a required additive for viral neuraminidase activity. For studies with H1N1, the nanogel was diluted to a final lipid concentration of 20% mass/volume using the aqueous background electrolyte for H1N1 separations (100 mM NaCl, 5 mM CaCl_2_, and 50 mM Tris buffered to pH 7.5). For studies with H5N1 the nanogel was diluted to a final lipid concentration of 25% mass/volume using the aqueous background electrolyte for H5N1 separations (100 mM NaCl, 5 mM CaCl_2_, and 50 mM MES buffered to pH 6.5). Nanogel preparations containing inhibitor were made by serial dilution of the inhibitor in the appropriate background electrolyte, with the final dilution step of 0.5 μL of aqueous inhibitor solution added to a 50 μL aliquot of nanogel. For all nanogel preparations, the final concentration of nanogel was 19.8% for H1N1 studies or 24.8% for H5N1 studies. The 5% nanogel used during capillary patterning for H1N1 was prepared by diluting 12.5 μL of the 20% nanogel stock with 38.0 μL of the aqueous background electrolyte. The 5% nanogel used during capillary patterning for H5N1 was prepared by diluting 10.0 μL of the 25% nanogel stock with 40.5 μL of the aqueous background electrolyte. For each inhibitor concentration analyzed, the inhibitor was included in the aqueous background electrolyte used to dilute nanogel and in the cathodic and anodic vials in contact with the platinum electrodes used to deliver high voltage. All phospholipid preparations were briefly subjected to a vortex mixer. Following mixing, bubbles were eliminated by freezing the solution in liquid nitrogen, allowing it to thaw to room temperature for approximately 10 min, and centrifuging the phospholipid preparation briefly (<1 min) with a mini-microcentrifuge (Cat 6765/C1501, Corning, LSE, Corning, NY) that delivers 6000 rpm (2000*g*).

Unless otherwise noted, 3 μL of the enzyme stock supplied by the manufacturer^14,15^ was diluted up to 10 μL to a final composition of 5% nanogel in 100 mM NaCl, 5 mM CaCl_2_, and 50 mM Tris buffered to pH 7.5 yielding 0.066 mg/mL H1N1 or in 100 mM NaCl, 5 mM CaCl_2_, and 50 mM MES buffered to pH 6.5 yielding 0.0701 mg/mL H5N1. The enzyme concentration is reported by the manufacturer as mg/mL rather than the activity based enzyme unit defined as Unit or U. For a 0.5 cm enzyme reaction zone (i.e., 0.4 nL volume in a 10 μm inner diameter capillary), this represents a total mass of 26 pg and 28 pg of H1N1 and H5N1 neuraminidase, respectively. The amount of enzyme (either H1N1 or H5N1) in the reaction zone was the same for each inhibition measurement. When not in use, enzyme was sealed and stored at 4 °C up to 2 weeks.

### Capillary Electrophoresis Assays

Separations were carried out on a P/ACE MDQ Plus (Sciex, Redwood City, CA) equipped with a laser-induced fluorescence detector using a 10 μm inner diameter 360 μm outer diameter fused-silica capillary (Polymicro Technologies, Phoenix, AZ). Each day before programming nanogel assays, the capillary was prepared with a flush sequence at 517 kPa (75 psi) with 1 M NaOH (30 min), deionized water (15 min), methanol (15 min), deionized water (15 min), background electrolyte (5 min), phospholipid coating (20 min), background electrolyte (5 min). The coating was composed of 5% (*q* = 0.5) phospholipid and used to passivate the charges on the capillary surface.^16−18^ The semipermanent lipid coating mitigated electroosmotic flow; thereby allowing separations of anionic compounds under conditions of reverse polarity. For analyses of H1N1 neuraminidase the background electrolyte is 50 mM TRIS, 5 mM CaCl_2_, and 100 mM NaCl buffered to pH 7.5. The 6′-sialyllactose substrate is made to a concentration of 26 nM in 1.5 mM TRIS buffered to pH 7.5. For analyses of H5N1 neuraminidase the background electrolyte is 50 mM MES, 5 mM CaCl_2_, and 100 mM NaCl buffered to pH 6.5. The substrate is made to a concentration of 200 nM in 1.5 mM MES buffered to pH 6.5. Substrate, enzyme, and nanogel were stored at 4 °C in a thermally regulated unit of the instrument. The data were collected using laser-induced fluorescence (λ_ex_ = 488 nm, λ_em_= 520 nm) and analyzed using 32 Karat Software version 10.2 (Sciex, Redwood City, CA). Enzyme activity measurements to evaluate the amount of product formed relative to substrate (i.e., peak area of product divided by the sum of peak areas of the substrate and the product), were expressed as the percent conversion. The effect of an inhibitor on the enzyme activity (i.e., percent activity remaining) was quantified as the ratio of the percent conversion in the presence of inhibitor to the percent conversion in the absence of inhibitor (e.g., see Table S1). The inhibition curves were fit using Graphpad Prism version 9.1.2 (Dotmatics, San Diego, CA) as the remaining enzyme activity vs log[Inhibitor]. The data were fit as a sigmoidal four parameter dose response curve constrained to converge at 0 and 100%.

Unless otherwise noted, the capillary was loaded with solutions as outlined below. Capillary flushing and filling were performed with the capillary temperature set to 15 °C. Prior to each separation the capillary was flushed for 5 min at 517 kPa (75 psi) using aqueous background electrolyte and then filled with 20% nanogel (*q* = 2.5) for 20 min at 517 kPa (75 psi). Next the *q* = 2.5, 5% nanogel zone (approximately 4 cm long) was introduced for 83.1 at 103 kPa (15 psi), followed by an enzyme zone (approximately 0.5 cm) for 11.1 s 103 kPa (15 psi), and then by a 20% nanogel zone (approximately 14.7 cm) for 540 s at 103 kPa (15 psi). After the enzyme was positioned in the capillary, mixing was done by pushing aqueous background electrolyte 12.1 s with 517 kPa (75 psi) in the reverse direction (from the detection to injection end) and then repeating the push using 20% nanogel 12.1 s with 517 kPa (75 psi) in the forward direction (from the injection to detection end). Once this patterning was complete, the APTS-labeled 6′-sialyllactose substrate was electrokinetically injected (−8 kV, 4s), followed by the injection of a post plug of nanogel for 13.9 s with 103 kPa (15 psi) to prevent the analyte from being ejected from the separation capillary. The temperature was then increased to 37 °C during a 5 min wait step, and the separation was accomplished at −12 kV. To prevent carryover of inhibitor or enzyme, an aqueous or nanogel dip step was implemented to wash the nanogel fill, or the enzyme off the electrodes and capillary ends.

## Results and Discussion

### Nanogel Assays

**Figure 1:**
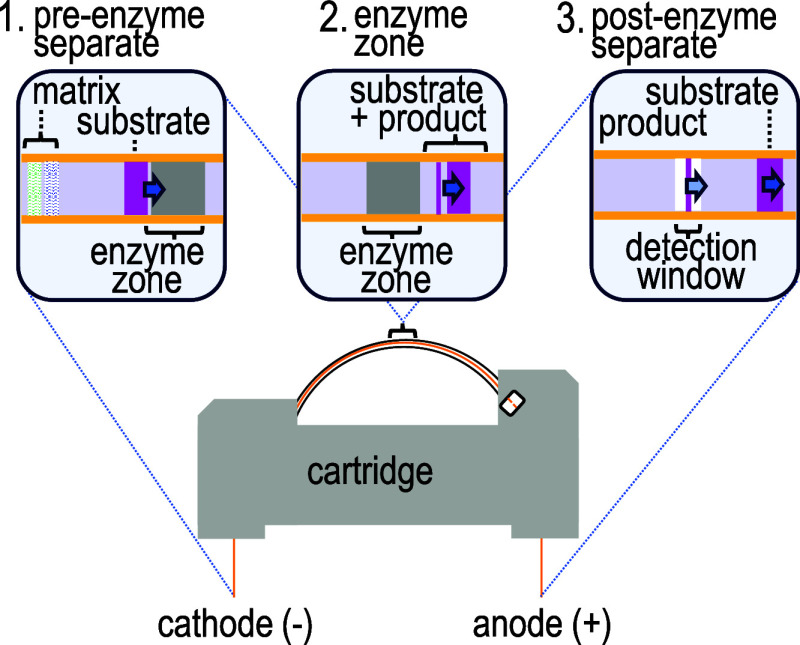
Illustrates the pre-enzyme reaction separation of the injected substrate (step 1), the enzymatic conversion (step 2) and the postenzyme reaction separation of the remaining substrate and newly formed product (step 3).

Gel media are used in capillary electrophoresis separations of glycans to more effectively suppress the electroosmotic flow in reversed polarity electrophoresis.^19^ Separations of oligosaccharides are improved by using gels. While gels serve to sort larger molecules based on electrophoretic sieving under conditions of minimal nonspecific binding to the analyte, they are also reported to create different degrees of compaction or coiling of the molecules based on the monomeric units composing the oligosaccharide.^20−22^ In addition to resolving the reaction components, nanogel was also used to define the region of the reaction zone. The neuraminidase enzyme hydrolyzed the APTS labeled-6′-sialyllactose to form the *N*-acetylneuraminic acid and lactose products. As summarized in Figure 1, this enzyme reaction was integrated in the electrophoresis capillary in order to include separation steps before and after the reaction zone. The region before the reaction zone separated interfering components in the matrix, such as the APTS dye and APTS labeled lactose byproducts that form as a result of spontaneous desialylation of the sialyllactose. Additionally, the region after the reaction zone separated and quantified the substrate and product following the reaction.

### Separations in a High Salt Background Electrolyte

Recombinant enzymes are often reconstituted in high salt concentrations to stabilize the structure. Capillary electrophoresis separations are typically developed with low ionic strength buffers to avoid Joule heating. The separation-based assay can be modified to reduce Joule heating by decreasing the capillary inner diameter to be compatible with high salt background electrolytes. In capillary zone electrophoresis separations with an unmodified fused silica capillary, increasing the ionic strength of the background electrolyte affects separations in other ways; for example, by decreasing the electroosmotic flow as well as the apparent hydrodynamic radius of the protein. Nanogel separations lessen these effects as the electroosmotic flow is suppressed and because the protein mobility is significantly reduced by sieving mechanisms induced by the presences of the viscous gel.

The tetrameric complex formed with recombinant H1N1 neuraminidase used in this work was composed of amino acid residues 37 to 469 from the 1918 Spanish flu virus neuraminidase (A/Brevig Mission/1/18) and was formulated in a high concentration of sodium chloride. A higher concentration of salt may stabilize the enzyme structure as well as shield electrostatic molecular interactions between the enzyme and substrate during the catalytic conversion. High salt concentrations have been reported to affect neuraminidase activity^23^ but not the inhibition of wild type neuraminidases.^24^ These effects of sodium chloride on recombinant tetrameric H1N1 neuraminidase could be evaluated by performing separation-based assays under a specific set of similar separation conditions which included a 25 μm inner diameter separation capillary, a separation voltage of −8 kV and a temperature at 20 °C. Assays performed in the absence and presence of 100 mM NaCl revealed a lower conversion by approximately 50% in the presence of salt (data and methods available in Figure S1 in the Supporting Information). Although the activity remaining was similar (47 ± 1% without salt vs 54 ± 2%) with salt, the inhibition was statistically different (student’s *t* test, *n* = 3, 95% confidence level). The preliminary studies in the 25 μm inner diameter capillary typically used for nanogel electrophoresis were adapted to a 10 μm inner diameter capillary to enable separations at higher voltages and at 37 °C.

**Figure 2:**
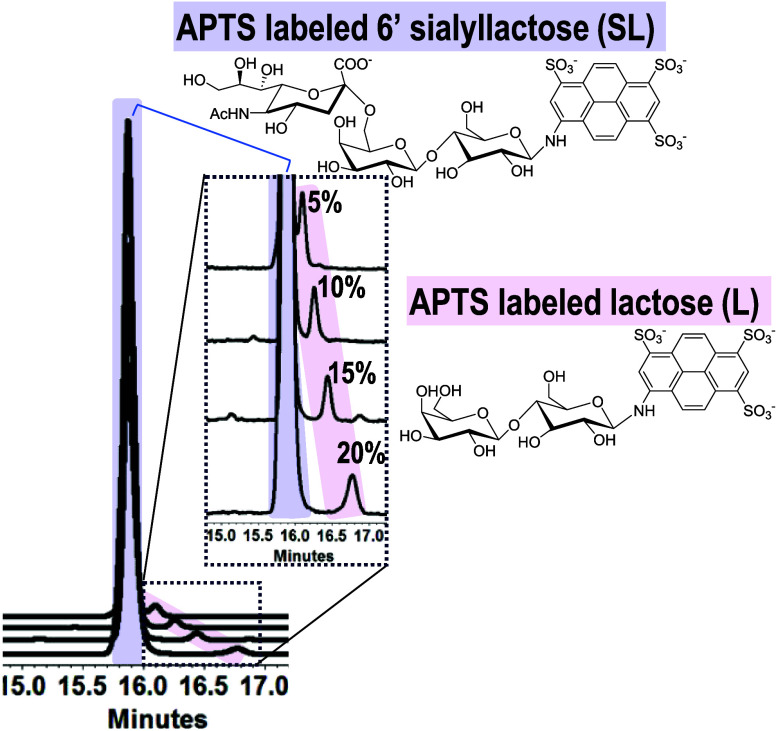
Shows the increase in resolution of 6′-siallylactose (SL) and lactose (L) with nanogel. Analytes are injected (−8 kV 4s) and separated in a 60 cm long 10 μm inner diameter capillary at −12 kV with a background electrolyte of 100 mM NaCl and 5 mM CaCl_2_ in 50 mM TRIS buffered to pH 7.5. Traces are offset for the purpose of visualization. The *x*-axis offsets from top to bottom are 0, −1.46, −3.52, −5.79 minutes. The *y*-axis offsets from top to bottom are 0, −0.06, −1.25, −0.20 RFU (see Tables S2A,B in the Supporting Information for data and calculations).

When using a background electrolyte composed of 100 mM sodium chloride, 5 mM CaCl_2_, 50 mM TRIS buffered to pH 7.5 the sialyllactose and lactose observed in a neuraminidase activity assay were not resolved in a 5% nanogel medium, requiring higher concentrations of nanogel additives. Although nanogels are compatible with high concentrations of sodium chloride,^25^ capillary nanogel electrophoresis has not previously been performed with nanogel formulated in high ionic strength buffers. Separations of sialyllactose and lactose using nanogels of varying concentrations formulated in 100 mM sodium chloride are shown in Figure 2. With increasing nanogel concentrations of 10, 15, and 20%, the resolution of the sialyllactose and lactose peaks increased to 2.6 ± 0.1, 3.3 ± 0.2, and 4.0 ± 0.1 (*n* = 3), respectively (see Table S2A,B in the Supporting Information). The improved separations at higher nanogel concentrations have been observed previously in the absence of sodium chloride^26^ and are attributed to the increased viscosity of nanogel at higher concentrations.^27,28^

### High Salt Nanogel Zones in a 10 μm Capillary

**Figure 3:**
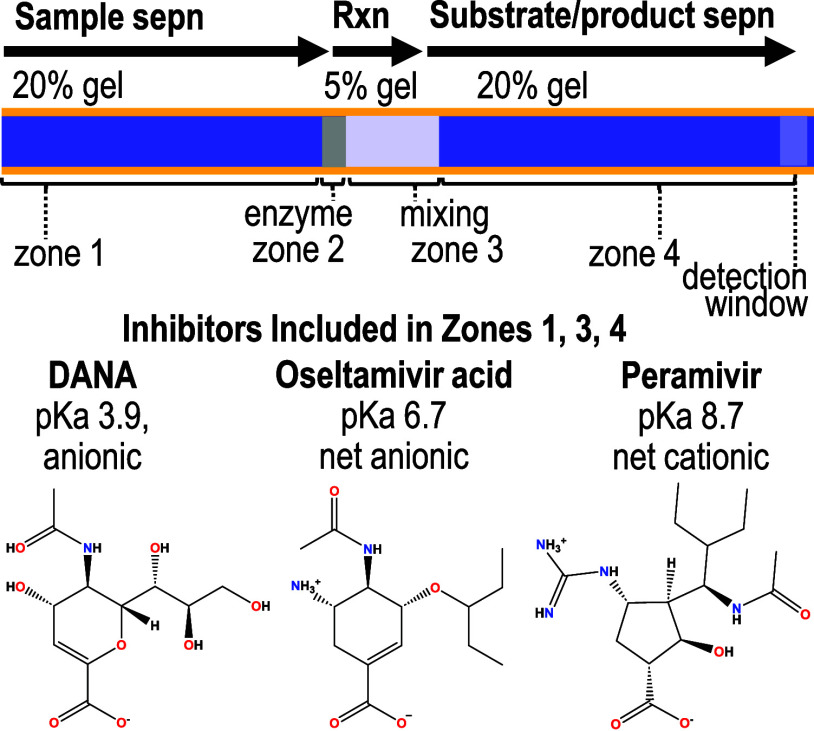
Depicts the zones created in a 60 cm long 10 μm inner diameter capillary, and the molecular structures for the inhibitors. The p*K*_a_ values are calculated using Advanced Chemistry Development (ACD/Laboratories) Software (1994–2024 ACD/Laboratories).

Nanogels were introduced into the capillary to create a pre-enzyme separation region (Figure 3, zone 1) to resolve the substrate from the matrix, an enzyme reaction region (Figure 3, zones 2,3), and a post enzyme reaction region (Figure 3, zone 4) to separate and quantify the substrate and product. The capillary was maintained at 15 °C during flushing and loading of the separation medium to keep the 20% nanogel in a low viscosity state in the capillary. Using the conditions outlined in the Materials and Methods Section the capillary was estimated to contain a preseparation, enzyme, and 5% zone length of 14.7, 0.5, and 4 cm, respectively. The objective of the nanogel electrophoresis patterning protocol was to mix inhibitor and enzyme in the capillary to eliminate manual mixing of the enzyme with each inhibitor in a separate vial. This is important because 5 μL is the minimum volume that can be introduced in a vial designed for the automated instrument. If manual mixing were required each point on the inhibition curve would require a 5 μL volume of enzyme. Although readily sourced, recombinant enzymes are available in limited volumes (e.g., 30 μL) and are often costly. With automated mixing, a 5 μL volume was required for the vial, but only a 0.4 nL volume was consumed in each separation. By mixing in the capillary, the enzyme consumption was kept low, and the automated instrument was used to create the inhibitor curves. Inhibitors were not a cost-limiting factor to the analyses, and were manually diluted to the desired concentration and a minimum volume of 5 μL of each inhibitor concentration was required for the assay.

The recombinant H1N1 neuraminidase which had an isoelectric point of 5.79 and a molecular weight of 48 kDa, was reconstituted in 5% nanogel buffered to pH of 7.5 as this pH was optimum for enzyme activity. In the pH 7.5 background electrolyte, this protein was anionic and migrated toward the detection window. In 20% nanogel, the electrophoretic mobility of the protein was slower than that of the anionic APTS-labeled sialyllactose in the 20% nanogel. Although the protein mobility was slow in 20% nanogel, where it exhibited size based sieving, it was faster in 5% nanogel where transport was based purely on electrophoresis (i.e., charge-to-size ratio). The use of 5% nanogel in zone 3 fostered mixing of the enzyme and inhibitor because it promoted movement of the enzyme from zone 2 to zone 3. In addition, the presence of the 5% nanogel increased enzyme conversion (see Table S3 in the Supporting Information) because it prevented sieving, thereby avoiding perturbations that deform the tetrameric form of the enzyme.

### Analysis of 1918 Pandemic Neuraminidase Activity

The inhibitors that were evaluated against H1N1 neuraminidase, shown in Figure 3, had different mobilities. To simplify the method, the inhibitors were added to the solutions in zones 1, 3, and 4, but not to the enzyme zone. As the background electrolyte was buffered to a pH of 7.5, the DANA and oseltamivir were anionic and migrated toward the detection window, whereas the peramivir was cationic and migrated toward the site of injection. The effectiveness of colocating the substrate and inhibitors with the enzyme was confirmed experimentally by performing the assay with inhibitor included throughout the capillary except for in the enzyme reaction zone.

The experiment confirming the colocation of inhibitor and enzyme involved comparing the activity remaining after patterning the capillary zones shown in Figure 3 with inhibitor in zones 1, 3, and 4 to the activity remaining after patterning the capillary zones shown in Figure 3 with inhibitor in zones 1, 2, 3, and 4. The concentrations of enzyme and substrate selected for the experiments resulted in the conversion of less than 10% of the substrate to product; thereby avoiding substrate depletion and maintaining the reaction at the initial enzyme velocity. The peak areas were used to determine the enzyme activity that remained in the presence of the inhibitor in two steps. First, the areas obtained for the substrate and product peaks were measured to derive the percent conversion which was calculated as the area of the product formed divided by the sum of the product and substrate areas. Second, the percent activity remaining was determined for each inhibitor concentration by dividing the percent conversion at a specific concentration by the percent conversion observed with no inhibitor present. Replicate runs were performed and normalized to matched assays performed in the absence of inhibitor to quantify the percent activity remaining. As summarized in Tables S4A–C in the Supporting Information, for the DANA, oseltamivir acid, and peramivir, the inhibition observed under both conditions was statistically the same (student’s *t* test, *n* = 3, 95% confidence level).

The sensitivity of the enzyme conversion to the amount of substrate in the reaction zone was evaluated. Enzyme conversion was measured with different concentrations of substrate. Although the peak areas differed, there was no statistical difference in the percent conversion (see Table S5A,B in the Supporting Information) observed at a 6′-sialyllactose concentration of 26 nM (3.4 ± 0.2%, *n* = 4) as compared to 200 nM (3.3 ± 0.3%, *n* = 4). Enzyme conversion was also evaluated when differences in the capillary patterning affected the amount of substrate that was introduced. The capillary was filled at 15 °C, which promotes the formation of anisotropic lipid bilayer disks exhibiting low viscosity. Then the substrate was injected and a nanogel post plug was introduced. The temperature of the cartridge was raised to 37 °C, which promotes the formation of entangled lipid ribbons and sheets exhibiting high viscosity. The introduction of a nanogel post plug following the injection was necessary because even a slight change in the temperature is known to induce a change in the nanogel morphology and in the molar expansion associated with the morphology change. The consequence of this is an expansion in molar volume which can inadvertently eject a portion of the substrate from the capillary^29^ before the substrate is electrophoretically driven deeper into the capillary. If a portion of the injected substrate is ejected from the capillary it affects the amount of substrate delivered to the enzyme, but it does not affect the percentage of the substrate that is converted to product in the enzyme zone. For example, as summarized in Table S6A,B (in the Supporting Information) decreasing the post plug that follows the substrate injection by 4 s decreased the area by half; yet the percent conversion was not statistically different (student’s *t* test, *n* = 4, 95% confidence level). Differences in the starting temperature of the nanogel preparation, in the ambient temperature during the wait period, or in the nanogel concentration affect the expansion process and can change the amount of substrate or nanogel introduced. If the nanogel reagents, stored in the refrigerated region of the instrument did not remain at the same temperature during the patterning steps for each measurement, the absolute peak areas of the substrate and product changed although the percent conversion was consistent.

### Assays of 1918 Pandemic Neuraminidase with DANA

**Figure 4:**
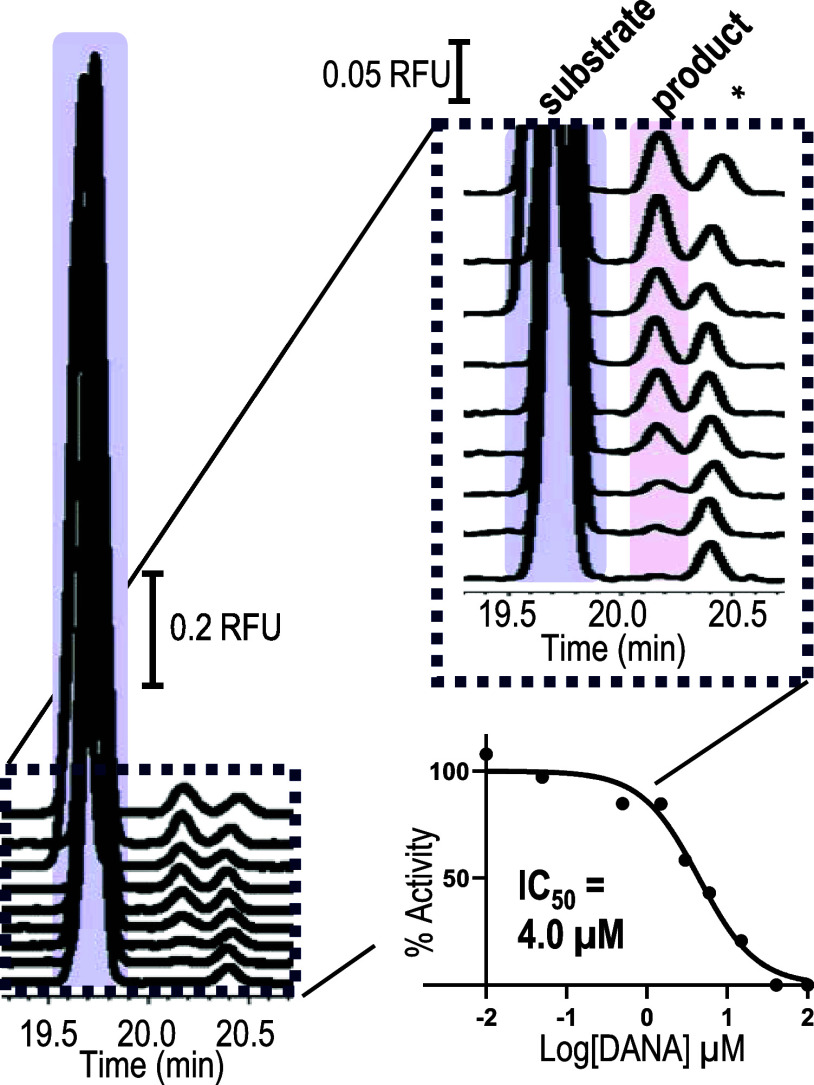
Depicts the traces obtained with H1N1 neuraminidase showing the conversion of the 6′-sialyllactose to lactose in the presence of the transition state analog DANA at concentrations from top to bottom as 0.010, 0.050, 0.50, 1.5, 3.0, 6.0, 15, 41, 100 μM. The product peak area decreases with increasing inhibitor. The *K_i_* is approximated as the IC_50_. Traces are offset to aid in visualization. See Table S7A and Figure S2A in the Supporting Information for peak areas, offsets for the *x*- and *y*-axes, and data for replicate traces. The peak labeled with the asterisk is a contaminant present in the substrate standard or sialyllactose that has undergone spontaneously desialylation during the APTS labeling reaction. This contaminant peak is typically about 1–2% of the peak area of the sialyllactose. As required for evaluating activity, the sialyllactose peak area is significantly larger than the product peak area.

The effect of an inhibitor on the enzyme activity was quantified by monitoring the conversion of the 6-sialyllactose substrate to the lactose product, as shown in Figure 4 and summarized in Table S7A in the Supporting Information. To obtain the inhibition constant, the effect of inhibitor on conversion was used to create a dose response curve of the percent activity that remained in the presence of the inhibitor at a range of concentrations. The IC_50_ value, which was the concentration at which 50% of the activity remained, was then converted to the *K_i_*. For these assays the IC_50_ value was equal to the *K_i_* as defined by *K_i_* = IC_50_/[1 + ([S]/*K*_m_)] because the concentration of the 6′sialyllactose substrate (i.e., [S]) is low relative to the Michaelis–Menten constant (*K*_m_).

**Figure 5:**
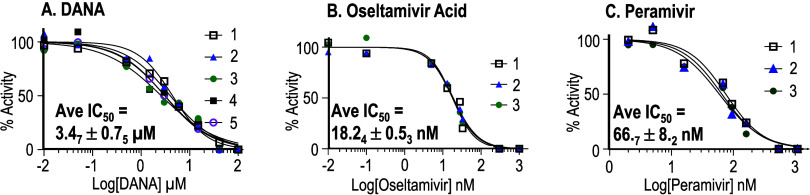
Dose response curves for viral neuraminidase inhibitors using 26.4 nM 6′-siallylactose. The *K_i_* is approximated as the IC_50_, which is reported in each graph as the average ± standard deviation. (A) DANA concentrations ranging from 10 nM to 100 μM, (B) oseltamivir acid concentrations ranging from 0.01 nM to 1 μM, and (C) peramivir concentrations ranging from 2.7 nM to 1.1 μM. See Tables S7–S9 and Figures S2–S4 in the Supporting Information for electropherograms, peak areas and inputs for each replicate curve.

This process was used to obtain a single curve, such as that depicted in Figure 4. Multiple curves were then obtained for each inhibitor. This was demonstrated in a set of 5 replicate curves with conditions that produce identical peak areas (i.e., curves 1,2) and with conditions that lead to differences in the peak areas (i.e., curves 3,4,5) but the activity remaining was consistent across all curves. The IC_50_ values of 5 replicate analyses of the inhibitory effect of DANA, summarized in Figure 5A, were statistically the same with an average *K_i_* = 3.5 ± 0.8 μm (see Tables S7A–E and Figures S2A–E in the Supporting Information).

### Assays of 1918 Pandemic Neuraminidase by Oseltamivir Acid and Peramivir

The utility of modern drugs to inhibit the H1N1 neuraminidase from the 1918 pandemic was also evaluated using the same patterning concept described for DANA. As shown in Figure 5B, oseltamivir acid, which is the active agent of the drug Tamiflu, resulted in a K_i_ value of 18.2 ± 0.5 nM (*n* = 3, IC_50_, electropherograms and data summarized in Tables S8A–C and Figure S3A–C in the Supporting Information). As shown in Figure 5C, peramivir, which is marketed as Rapivab, resulted in a *K_i_* value of 67 ± 8 nM (*n* = 3, IC_50_, electropherograms and data summarized in Tables S9A–C and Figure S4A–C in the Supporting Information). It is difficult to directly compare these *K_i_* values to literature values because of the limited access, and risks associated, with the use of viable virus specimens that are sourced from the 1918 pandemic. However, pharmaceutical efficacy can be evaluated by ranking *K_i_* values.^30^ Lower *K_i_* values are considered to have more promising therapeutic potential. As anticipated, the *K_i_* values of oseltamivir acid and peramivir were significantly lower than that observed for the transition state analog DANA.

Although a direct literature comparison of H1N1 neuraminidase collected in the 1918 pandemic cannot be made with *K_i_* values, the IC_50_ values for DANA, oseltamivir acid, and peramivir can be benchmarked against the H1N1 virus collected in 1934 from Puerto Rico (A/PR/8/34), which is a strain that evolved from the 1918 virus. An assay reported in the literature used intact virus particles to stabilize the tetrameric enzyme structure of A/PR/8/34 neuraminidase with a cell viability evaluation using neutral red following incubation with MDCK cells.^31^ A second report used a MUNANA assay in a low salt formulation.^32^ The IC_50_ value was comparable to DANA (1 μM).^32^ Similar to the trend observed with the capillary nanogel electrophoresis measurements, the EC_50_ values reported for oseltamivir (220 nM) and peramivir (1.5 μM)^31^ reflect the stronger therapeutic effectiveness of oseltamivir over peramivir.

### Inhibition Assays of 2004 Pandemic H5N1 Neuraminidase

The capillary electrophoresis- based assay was further adapted to use with a different pandemic influenza A virus. H5N1 strains are of particular interest at present because they originate as an avian virus selective for end-capped glycosylation presenting α2–3 linked sialylated compounds predominant in birds, that ultimately adapt to the α2–6 linked sialylated compounds prevalent in humans. The more common MUNANA assay does not distinguish linkage specificity because the substrate lacks the sialic acid-galactose sequence. Applying the capillary electrophoresis inhibition assay to the H5N1 recombinant protein incorporated a substrate that reflected the α2–6-sialylation that was relevant to virulence in humans. A recombinant protein based on the 2004 pandemic strain A/Vietnam/1203/2004 H5N1 which has a *K*_m_ of 214 μM,^33^ forms a tetrameric compound; however, the optimum pH for H5N1 assays is 6.5.^15,33^

**Figure 6:**
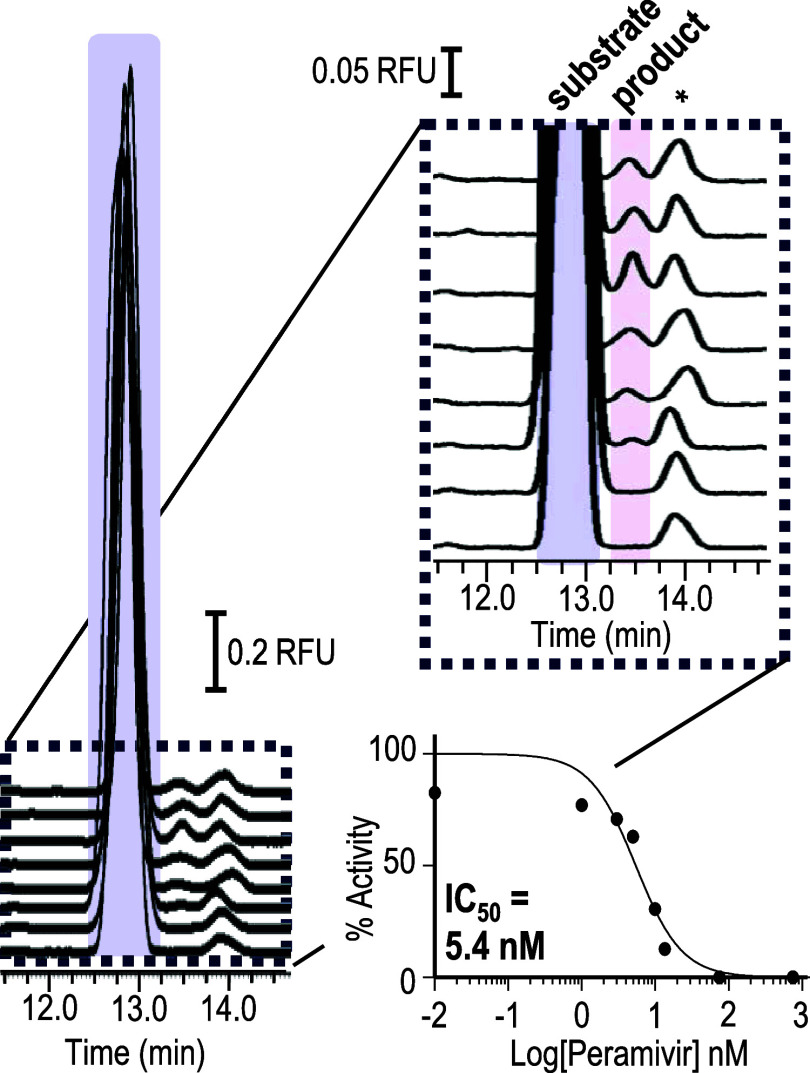
Depicts the traces obtained with H5N1 neuraminidase showing the conversion of the 6′-sialyllactose substrate to the lactose product in the presence of peramivir at concentrations listed from top to bottom as 0.010, 1.0, 3.0, 5.0, 10., 14, 75, 750 nM. The area of the product, which decreases with increasing inhibitor, is used to create the dose response curve. The *K_i_* is approximated as the IC_50_. Traces are offset to aid in visualization (see Table S13, and Figure S7 in the Supporting Information for peak areas and offsets for the *x*- and *y*-axes). The peak labeled with the asterisk is a contaminant present in the substrate preparation prior to enzyme treatment.

The separation method was modified to resolve the substrate and product (Figure 6) with a background electrolyte containing 100 mM NaCl to preserve the tetrameric structure and the pH of 6.5 to maintain maximum enzyme conversion. To achieve this resolution, a 25% nanogel was used (see Figure S5 and Tables S10A,B in the Supporting Information). The effect of high salt on the activity and inhibition was verified (see Table S11A,B and Figure S6A,B in the Supporting Information). The enzyme patterning in a 10 μm inner diameter capillary was similar, and an experiment confirming the colocation of inhibitor and enzyme was performed (see Table S12 in the Supporting Information) to validate the use of in-capillary mixing of enzyme and inhibitor.

The recombinant enzyme for A/Vietnam/1203/2004 H5N1 is based on residues 37–449 of the wild type neuraminidase which was modified to also contain an N-terminal vasodilator-stimulated phosphoprotein tetramerization domain and a C-terminal 6-His tag. The inhibition of the recombinant H5N1 viral neuraminidase was evaluated with peramivir with an IC_50_ curve constructed with 8 inhibitor concentrations. The results, summarized in Figure 6, yielded a *K_i_* of 5.4 nM for peramivir (data and methods available in Figure S7 and Table S13 in the Supporting Information). The inhibition observed by peramivir can be compared to a literature IC_50_ value of 0.6 nM reported for wild type A/Vietnam/1203/2004 H5N1, which was obtained with a fluorometric MUNANA assay at an unspecified pH value and salt concentration.^34^ Although the substrate and reaction conditions differ, the capillary nanogel electrophoresis assay demonstrated the activity of a recombinant enzyme containing a tetramerization domain foreign to H5N1 neuraminidase.

## Conclusions and Future Directions

Advances in biotechnology provide the means to rapidly identify viral genomic sequences and to recreate recombinant proteins. Access to recombinant multimeric proteins enables researchers to evaluate enzyme activity and inhibition without concern for biological containment that is required when working with live viruses. However, assays developed for recombinant neuraminidase from influenza must preserve the activity of the tetrameric structure. The capillary electrophoresis enzyme assay outlined in this report is the first example of a high salt nanogel separation and was possible by reducing the capillary inner diameter to 10 μm. Traditional sieving gels are difficult to use with narrow bore capillary; however, the thermally switchable viscosity of nanogels made the assay possible. The capillary nanogel electrophoresis was an effective means to analyze recombinant enzymes from viral neuraminidase present in H1N1 and H5N1 influenza A that led to pandemics in 1918 and 2004. The approach consumed as little as 0.4 nanoliter of enzyme per run and was fully automated using commercially available electrophoresis instrumentation. With the in-capillary patterning, the substrate was separated from contaminants and APTS dye prior to the enzymatic hydrolysis while the reaction product was separated from the substrate. The consumption of substrate and enzyme was small, allowing for routine use of substrate that contained sialic acid residues with linkage positions that are relevant to viral infections and the adaptation of viruses, such as H5N1, from linkages associated with different species (e.g., birds vs humans). The method reported here can be applied to any viral neuraminidase to evaluate the effectiveness of therapeutics that are currently approved to treat influenza A. The method can also be used to quantify the efficacy of new neuraminidase inhibitors and to determine the specificity of an enzyme for different sialylated substrates. Beyond neuraminidases, the capillary nanogel electrophoresis assay can be adapted to different enzymes and substrates. Future work involves the evaluation of other recombinant multimeric enzymes and a wider array of physiologically relevant substrates.

## Supporting Information Available

Electropherograms and data from experiments associated with *K_i_* measurements and method development (PDF)
