# Correction to “Native Capillary Nanogel Electrophoresis Assay of Inhibitors of Neuraminidases Derived from H1N1 and H5N1 Influenza A Pandemics”

**DOI:** 10.1021/acs.analchem.5c01608

**Published:** 2025-03-31

**Authors:** Laura N. Taylor, Lisa A. Holland, Makenzie T. Witzel

Corrections are reported for the Supporting Information in our published article reflecting two peak areas which were mistyped, revised data displaying only significant digits in means and standard deviations, and an amended computation in Table S11A. The revised Supporting Information file is given here.

Also, corrections are reported for the published article reflecting two values.

In the abstract, on page 5082 and in Figure 5A: For 1918 H1N1 (A/Brevig Mission/1/18) neuraminidase, the inhibition constant of the transition state analog 2,3-dehydro-2-deoxy-N-acetylneuraminic acid (DANA) is 3.4 μM, which is derived from the corrected value of 3.4_4_ ± 0.7_6_.

On page 5080: The H1N1 % enzyme activity remaining in the presence of sodium chloride has a standard deviation of ± 1.

## Supporting Information Available

Electropherograms and data from experiments associated with *K*_*i*_ measurements and method development (PDF)

