# Supplementary material for: Native Capillary Nanogel Electrophoresis Assay of Inhibitors of Neuraminidases Derived from H1N1 and H5N1 Influenza A Pandemics
Source: Anal Chem. 2025 Feb 28;97(9):5077–84. doi: 10.1021/acs.analchem.4c06127 (PMC11912125; doi:10.1021/acs.analchem.4c06127)
Supplement: Supplementary file 1 — ac4c06127_si_001.pdf [file ac4c06127_si_001.pdf]

SUPPORTING INFROMATION: NATIVE CAPILLARY NANOGEL ELECTROPHORESIS ASSAY OF INHIBITORS OF NEURAMINIDASES DERIVED FROM H1N1 AND H5N1 INFLUENZA A PANDEMICS

Laura N. Taylor, Lisa A. Holland, and Makenzie T. Witzel

[1]C. Eugene Bennett Department of Chemistry, West Virginia University, Morgantown, WV 26505

ABSTRACT

This material includes electropherograms and tabular data from experiments demonstrating high salt nanogel separations zone, enzyme conversion, inhibitor introduction and replicates associated K_i experiments. Original data available through Mendeley Data: Holland, Lisa (2025), “Data for: Native Capillary Nanogel Electrophoresis Assay of Inhibitors of Neuraminidases Derived from H1N1 and H5N1 Influenza A Pandemics”, Mendeley Data, V1, doi: 10.17632/wnzc246cmw.1

TABLE OF CONTENTS

Page S1 Abstract, Table of contents

Page S2 Table of contents, Authorship credit statement

Page S3 Table S1

Page S4 Figure S1, Effect of 100 mM NaCl on neuraminidase conversion

Page S5 Table S2A,S2B Resolution achieved with increasing nanogel concentration

Page S6 Table S3. The effect of patterning 5% with DANA

Page S7 Table S4A DANA zone study

Page S8 Table S4B oseltamivir acid zone study

Page S9 Table S4C peramivir zone study

Page S10 Table S5A. Varying [6’-SL] to Investigate changes in % Conversion, Table S5B Summary of Table S5A

Page S11 Table S6A. Altering post fill plug injection changes SL area, Table S6B Summary of Table S6A

Page S12 Table S7A DANA IC_{50} Curve #1 results and dose-response inputs, Figure S2A Curve 1 traces

Page S13 Table S7B DANA IC_{50} Curve #2 results and dose-response inputs, Figure S2B Curve 2 traces

Page S14 Table S7C DANA IC_{50} Curve #3 results and dose-response inputs, Figure S2C Curve 3 traces

Page S15 Table S7D DANA IC_{50} Curve #4 results and dose-response inputs, Figure S2D Curve 4 traces

Page S16 Table S7E DANA IC_{50} Curve #5 results and dose-response inputs, Figure S2E Curve 5 traces

Page S17 Table S8A. Oseltamivir Acid IC_{50} Curve #1 results and dose-response inputs, Figure S3A Curve 1 traces

Page S18 Table S8B. Oseltamivir Acid IC_{50} Curve #2 results and dose-response inputs, Figure S3B Curve 2 traces
Page S19 Table S8C. Oseltamivir Acid IC_{50} Curve #1 results and dose-response inputs, Figure S3C Curve 3 traces
Page S20 Table S9A. Peramivir IC_{50} Curve #1 results and dose-response inputs, Figure S4A Curve 1 traces
Page S21 Table S9B. Peramivir IC_{50} Curve #2 results and dose-response inputs, Figure S4B Curve 2 traces
Page S22 Table S9C. Peramivir IC_{50} Curve #3 results and dose-response inputs, Figure S4C Curve 3 traces
Page S23 Table S10A Resolution achieved with increasing nanogel concentration, Table S10B Summary of Table S10A
Page S24 Figure S5. Traces depicting resolution achieved with increasing nanogel concentration
Page S25 Table S11A. Effect of 0 mM NaCl on H5N1 Neuraminidase

Page S26 Figure S6A Traces depicting effect of 0 mM NaCl on H5N1

Page S27 Table S11B. Effect of 100 mM NaCl on H5N1 Neuraminidase, Figure S6B Traces depicting effect of 100 mM NaCl on H5N1
Page S28 Table S12 Peramivir zone study

Page S29 Table S13. Peramivir H5N1 IC_{50} Curve results and dose-response inputs, Figure S7 Curve traces

CRediT authorship contribution statement based on Brand, A.; Allen, L.; Altman, M.; Hlava, M.; Scott, J. Beyond Authorship: Attribution, Contribution, Collaboration, and Credit. Learned Publishing 2015, 28 (2), 151-155. DOI: 10.1087/20150211.

LNT: Methodology, Validation, Formal Analysis, Investigation, Data Curation Writing- Original Draft preparation, Writing – Review and Editing, Visualization. **LAH:** Conceptualization, Methodology, Investigation, Writing- Original Draft preparation, Writing – Review and Editing, Visualization, Supervision, Project Administration, Funding Acquisition. **MTW**: Methodology.

Table S1. Effect of 100 mM NaCl on H1N1 Neuraminidase Conversion

	[DANA][a]	6'-SL Area[a]	Lactose Area	Total Area	Conversion (%)[b]	Activity Remaining (%)[c]
0 mM NaCl	No Inhibitor	4475736	64995	4540731	1.43	100
		4695627	81080	4776707	1.70	100
		4653954	79419	4733373	1.68	100
	***Average Conversion**: $1.6_0 \pm 0.1_4$ (9% RSD)*					
	1 μM	4866239	35922	4902161	0.73	45.74
		5024033	39369	5063401	0.78	48.53
		5050518	38386	5088904	0.75	47.08
	***Average % Activity Remaining**: $47._1 \pm 1._3$ (3% RSD)[d]*					
100 mM NaCl	No Inhibitor	10964532	85754	11050286	0.78	100
		11283796	96709	11380505	0.85	100
		10437696	86381	10524077	0.82	100
	***Average Conversion**: $0.81_6 \pm 0.03_7$ (5% RSD)*					
	1 μM	11911443	53981	11965424	0.45	55.32
		11885712	52496	11938208	0.44	53.92
		10375981	44520	10420501	0.43	52.39
	***Average Activity Remaining**: $53._8 \pm 1._5$ (3% RSD)[d]*					

[a] *Abbreviations: DANA 2,3-dehydro-2-deoxy-N-acetylneuraminic acid (DANA), 6'-sialyllactose (6'-SL)*
[b] *The percent enzyme conversion is calculated as the area of lactose divided by total area.*
[c] *The percent activity remaining is calculated as the percent conversion with inhibitor divided by the average percent conversion without inhibitor.*
[d] *The averages of percent activity remaining for 0 mM NaCl and 100 mM NaCl are statistically different (student's t-test, n = 3, 95 % confidence level).*

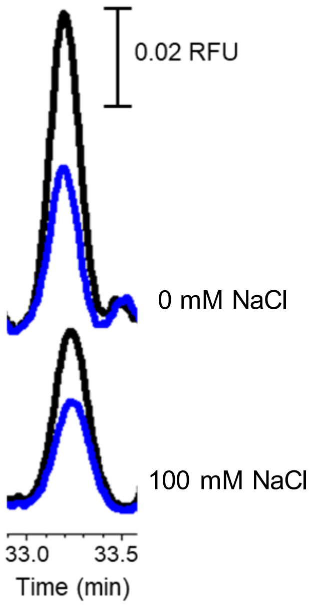

Figure S1 summarizes the effect of 100 mM sodium chloride on the enzyme activity and the inhibition for H1N1 neuraminidase. The separations are performed in the absence (black traces) and presence (blue traces) of 1 μM 2,3-dehydro-2-deoxy-*N*-acetylneuraminic acid (DANA) in a 25 μm inner diameter capillary. The top traces contain no salt whereas the bottom traces contain 100 mM NaCl. For the purpose of visualization, some traces are offset, and for 100 mM NaCl traces are scaled. For traces obtained with 0 mM NaCl, the black and blue traces were offset in the x-axis by 0.0 and -0.39 min, respectively and in the y-axis by 0.0 and 0.003 RFUs, respectively. For traces obtained with 100 mM NaCl, the black and blue traces were scaled by 0.5 in the y-axis and offset in the x-axis by -1.85 min, and -2.65 min, respectively and in the y-axis by -0.015 and -0.018 RFUs, respectively. The H1N1 salt study required a 25 μm inner diameter capillary and a separation at 20 °C for baseline resolution of the product peak with the background electrolyte (BGE) containing either 0 mM or 100 mM NaCl. Patterning was done at 15 °C. The capillary was flushed for 6 min at 172 kPa (25 psi) with the BGE composed of 50 mM TRIS, 5 mM CaCl2 at pH 7.5 and either 0 mM or 100 mM NaCl, and then for 10 min 172 kPa (25 psi) with 20% nanogel fill. Next a 5% nanogel zone was introduced at 48 kPa (7 psi) for 21.3 s followed by an enzyme zone for 4.9 s at 34 kPa (5 psi). The 0.2 cm enzyme zone was then pushed 23 cm with 20% nanogel for 273.3 s at 48 kPa (7 psi). Next mixing in the capillary was done by pushing BGE at 48 kPa (7 psi) for 24.3 s in the reverse direction and then pushing 20% nanogel at 48 kPa (7 psi) for 24.3 s in the forward direction. Once this patterning was complete, the sample was electrokinetically injected (-8kV, 4s), followed by the injection of a post plug of 20% nanogel for 6.1 s with 48 kPa (7 psi) to prevent the analyte from being ejected from the separation capillary. The capillary temperature was raised to 20 °C during a 5-minute wait step. The separation was done with an applied voltage of -8 kV. To prevent contamination, the electrodes and capillary ends were dipped in BGE before and after the introduction of enzyme.

Table S2A. Resolution Achieved with Increased Nanogel Concentration

	% Nanogel	6'-Siallylactose Time (min)	6'-Siallylactose WHM[a] (min)	Lactose Time (min)	Lactose WHM (min)[a]	Resolution[b]
Set 1	5	15.283	0.077	15.512	0.101	1.52
	10	16.883	0.082	17.267	0.086	2.70
	15	18.550	0.096	19.154	0.105	3.55
	20	20.825	0.114	21.671	0.133	4.04
Set 2	5	15.871	0.082	16.100	0.098	1.50
	10	17.342	0.086	17.717	0.092	2.49
	15	19.400	0.096	19.954	0.101	3.32
	20	21.650	0.122	22.563	0.144	4.05
Set 3	5	16.462	0.084	16.679	-[c]	-[c]
	10	18.058	0.088	18.438	0.091	2.51
	15	20.642	0.106	21.233	0.118	3.11
	20	23.842	0.180	25.087	0.205	3.82

[a] *Width at half maximum height (WHM)*
[b] *Resolution is calculated as 1.18*(time lactose- time sialyllactose)/(WHM lactose + WHM sialyllactose).*
[c] *The WHM cannot be determined using automatic peak detection with the data processing software.*

Table S2B. Summary of Resolution Data in Table S2A

% Nanogel	Resolution (n = 3)
**5**	1.5[a]
**10**	$2.5_6 \pm 0.1_2$ (5% RSD)
**15**	$3.3_3 \pm 0.2_2$ (7% RSD)
**20**	$3.9_7 \pm 0.1_3$ (3% RSD)

[a] *Calculated from n=2*

Table S3. The Effect of Patterning 5% with DANA[a]

[DANA][a]	6'-SL Area[a]	Lactose Area	Total Area	% Conversion[b]	% Activity Remaining[c]
**No 5% Nanogel included in Pattern**					
No Inhibitor	624534	32211	656745	4.90	100
	614241	30388	644629	4.71	100
	706596	37888	744484	5.09	100
***Average % Conversion**: $4.9_{0} \pm 0.1_{8}$ % (4% RSD)[d]*					
3 µM	682369	17456	699825	2.49	50.88
	560643	17520	578163	3.03	61.81
	521397	16024	537421	2.98	60.82
***Average Activity Remaining:** $57._{8} \pm 6._{0}$ % (10% RSD)[e]*					
**5% Nanogel included in Pattern**					
No Inhibitor	639991	38219	678210	5.64	100
	642027	37951	679978	5.58	100
	680645	44762	725407	6.17	100
***Average % Conversion:** $5.8_{0} \pm 0.3_{3}$ % (6% RSD)[d]*					
3 µM	769311	20425	789736	2.59	44.62
	789244	27986	817230	3.42	59.09
	752558	24661	777219	3.17	54.75
***Average Activity Remaining:** $52._{8} \pm 7._{4}$ % (10% RSD)[e]*					

[a]Abbreviations: DANA 2,3-dehydro-2-deoxy-N-acetylneuraminic acid (DANA), 6'-sialyllactose (6'-SL)
[b]The percent enzyme conversion is calculated as the area of lactose divided by total area.
[c]The percent activity remaining is calculated as the percent conversion with inhibitor divided by the average percent conversion without inhibitor.
[d]The average conversion is statistically different in the absence and presence of 5% nanogel (student's t-test, n = 3, 95 % confidence level).
[e]The average percent activity remaining is statistically the same in the absence and presence of 5% nanogel (student's t-test, n = 3, 95 % confidence level).

Table S4A. Zone Study of DANA[a]

	6'-SL Area[a]	Lactose Area	Total Area	% Conversion[b]	% Activity Remaining[c]
**Set 1 Patterning with 0 μM DANA Inhibitor in the Enzyme Stock (Zone 2)**					
No Inhibitor 1	695482	38665	734147	5.27	100.00
Inhibitor in zones 1, 3, and 4	774607	29020	803627	3.61	68.57
	732035	28276	760311	3.72	70.61
	685861	29518	715379	4.13	78.35
Average Activity Remaining: $72._{5} \pm 5._{1}$ (7% RSD)[d]					
**Set 2 Patterning with 3 μM DANA Inhibitor in the Enzyme Stock (Zone 2)**					
No Inhibitor 2	653877	45634	699511	6.52	100.00
Inhibitor in zones 1, 2, 3, and 4	706407	24160	730567	3.31	50.69
	708419	27993	736412	3.80	58.27
	686604	33505	720109	4.65	71.32
Average Activity Remaining: $60._{0} \pm 10._{4}$ *(20% RSD)*[d]					

[a] *Abbreviations: DANA 2,3-dehydro-2-deoxy-N-acetylneuraminic acid (DANA), 6'-sialyllactose (6'-SL). A 400 s push step and an additional 200 s 103 kPa (15 psi) 20% nanogel post plug were used after the enzyme zone was patterned to mitigate the effects of nanogel expansion.*

[b] *The percent enzyme conversion is calculated as the area of lactose divided by total area.*

[c] *The percent activity remaining for set 1 vs. set 2 is calculated as the percent conversion with inhibitor divided by the percent conversion without inhibitor. The runs obtained in the absence of inhibitor for set 1 (No Inhibitor 1) and for set 2 (No Inhibitor 2) were done immediately before the set of replicate runs and used to normalize the percent conversion for each set.*

[d] *The average percent activity remaining with inhibitor in zones 1, 3, and 4 is statistically the same as that obtained with inhibitor in zones 1, 2, 3, and 4 (student's t-test, n = 3, 95 % confidence level).*

Table S4B. Zone Study of Oseltamivir Acid[a]

	6'-SL Area[b]	Lactose Area	Total Area	% Conversion[c]	% Activity Remaining[d]
**Set 1 Patterning with 0 nM Oseltamivir Acid Inhibitor in the Enzyme Stock (Zone 2)**					
No Inhibitor 1	1676202	48886	1725088	2.83	100.00
Inhibitor in zones 1, 3, and 4	1445748	22375	1468123	1.52	53.78
	1273180	23093	1296273	1.78	62.87
	1637451	30445	1667896	1.83	64.41
Average Activity Remaining: 60.3 ± 5.7 (10% RSD)[e]					
**Set 2 Patterning with 23 nM Oseltamivir Acid Inhibitor in the Enzyme Stock (Zone 2)**					
No Inhibitor 2	1378126	55534	1433660	3.87	100.00
Inhibitor in zones 1, 2, 3, and 4	1175277	26392	1201669	2.20	56.70
	1176301	28019	1204320	2.33	60.06
	1088437	27799	1116236	2.49	64.29
Average Activity Remaining: 60.3 ± 3.8 (6% RSD)[e]					

[a] *For this study 5 μL of the enzyme stock supplied by the manufacturer was diluted up to 10 μL to a final composition of 5% nanogel in 100 mM NaCl, 5 mM CaCl2, and 50 mM Tris buffered to pH 7.5 yielding 0.11 mg/mL H1N1*

[b] *Abbreviation: 6'-sialyllactose (6'-SL)*

[c] *The percent enzyme conversion is calculated as the area of lactose divided by total area.*

[d] *The percent activity remaining for set 1 vs. set 2 is calculated as the percent conversion with inhibitor divided by the percent conversion without inhibitor. The runs obtained in the absence of inhibitor for set 1 (No Inhibitor 1) and for set 2 (No Inhibitor 2) were done immediately before the set of replicate runs and used to normalize the percent conversion for each set.*

[e] *The average percent activity remaining with inhibitor in zones 1, 3, and 4 is statistically the same as that obtained with inhibitor in zones 1, 2, 3, and 4 (student's t-test, n = 3, 95 % confidence level).*

Table S4C. Zone Study of Peramivir					
	6'-SL Area[a]	Lactose Area	Total Area	% Conversion[b]	% Activity Remaining[c]
**Set 1 Patterning with 0 nM Peramivir Inhibitor in the Enzyme Stock (Zone 2)**					
No Inhibitor 1	1444514	20533	1465047	1.40	100.00
Inhibitor in zones 1, 3, and 4	1477388	15822	1493210	1.06	75.60
	1372309	15143	1387452	1.09	77.87
	1297081	15084	1312165	1.15	82.02
Average Activity Remaining: 78.5 ± 3.2 (4% RSD)[d]					
**Set 2 Patterning with 16 nM Peramivir Inhibitor in the Enzyme Stock (Zone 2)**					
No Inhibitor 2	1435546	24088	1459634	1.65	100.00
Inhibitor in zones 1, 2, 3, and 4	1271472	14447	1285919	1.12	68.08
	1225498	15447	1240945	1.24	75.43
	1241341	16335	1257676	1.30	78.70
Average Activity Remaining: 74.0 ± 5.4 (5% RSD)[d]					

[a] Abbreviation: 6'-sialyllactose (6'-SL)
[b] The percent enzyme conversion is calculated as the area of lactose divided by total area.
[c] The percent activity remaining for set 1 vs. set 2 is calculated as the percent conversion with inhibitor divided by the percent conversion without inhibitor. The runs obtained in the absence of inhibitor for set 1 (No Inhibitor 1) and for set 2 (No Inhibitor 2) were done immediately before the set of replicate runs and used to normalize the percent conversion for each set.
[d] The average percent activity remaining with inhibitor in zones 1, 3, and 4 is statistically the same as that obtained with inhibitor in zones 1, 2, 3, and 4 (student's t-test, n = 3, 95 % confidence level).

Table S5A. Effect of 6'-SL Concentration on Conversion[a]

	[6'-SL] nM	6'-SL Area	Lactose Area	Total Area	Conversion (%)[b]
Set 1	26	893575	29588	923163	3.21
	200	5510063	166783	5676846	2.94
Set 2	26	848271	28992	877263	3.30
	200	6529284	204338	6733622	3.03
Set 3	26	839410	29987	869397	3.45
	200	5495406	202696	5698102	3.56
Set 4	26	874625	33790	908415	3.72
	200	5848298	218632	6066930	3.60

[a] *Abbreviation: 6'-sialyllactose (6'-SL). SL is reconstituted in 1.5 mM TRIS buffered to pH 7.5.*
[b] *The percent enzyme conversion is calculated as the area of lactose divided by total area.*

Table S5B. Summary of Table S5A

[6'-SL] in nM[a]	Conversion (%)[b]
26	$3.4_2 \pm 0.2_2$ (7% RSD)
200	$3.2_8 \pm 0.3_4$ (10% RSD)

[a] *Abbreviation: 6'-SL 6'-sialyllactose*
[b] *The percent enzyme conversion is calculated as the area of lactose divided by total area. The average conversion with 26 nM 6'-SL is statistically the same as that obtained 200 nM 6'-SL (student's t-test, n = 4, 95 % confidence level).*

Table S6A. Effect of Post Injection Plug Size on Area and Conversion

	Post injection plug time (seconds)[a]	6'-SL Area[b]	Lactose Area	Total Area	Conversion (%)[c]
Set 1	17.9	694372	10627	704999	1.51
	13.9	788574	15011	803585	1.87
	9.90	414203	7996	422199	1.89
Set 2	17.9	756224	14801	771025	1.92
	13.9	730246	14596	744842	1.96
	9.90	330055	7072	337127	2.10
Set 3	17.9	809137	16466	825603	1.99
	13.9	734773	16289	751062	2.17
	9.90	491506	10192	501698	2.03
Set 4	17.9	698710	15086	713796	2.11
	13.9	749709	14685	764394	1.92
	9.90	375906	7691	383597	2.00

[a] *After the sample injection is complete a post plug of nanogel is introduced at 103 kPa (15 psi) for the times specified in the Table.*

[b] *Abbreviation: 6'-sialyllactose (6'-SL)*

[c] *The percent enzyme conversion is calculated as the area of lactose divided by total area.*

Table S6B Summary of Post Fill Plug Injections in Table S6A

Post injection plug time (seconds)	6'-SL Area x 10^5 (*n*=4)[a,b]	Conversion (%) (*n*=4)[c]
17.9	$7.4_0 \pm 0.5_4$ (7%)	$1.8_8 \pm 0.2_6$ (10% RSD)
13.9	$7.5_1 \pm 0.2_7$ (4%)	$1.9_8 \pm 0.1_3$ (7% RSD)
9.9	$4.0_3 \pm 0.6_8$ (20%)	$2.00_7 \pm 0.08_5$ (4% RSD)

[a] *Abbreviation: 6'-sialyllactose (6'-SL)*

[b] *The average peak area at 17.9 s is statistically the same as that obtained 13.9 s (student's t-test, n = 4, 95 % confidence level). The average peak area at 9.9 s is statistically different from that obtained 13.9 s or at 17.9 s (student's t-test, n = 4, 95 % confidence level).*

[c] *The percent enzyme conversion is calculated as the area of lactose divided by total area. The average percent conversions obtained at 17.9 s, 13.9 s, and 9.9 s are statistically the same (student's t-test, n = 4, 95 % confidence level).*

Table S7A. DANA IC$_{50}$ Curve #1 Results and Dose-response Inputs[a]

log[Inhibitor] (x-axis)		% Activity (y-axis input)				
[DANA] (μM)	**log[DANA] (μM)**	6'-SL Area	Lactose Area	Total Area	Conversion (%)	**Remaining Activity (%)**
0.010	-2.00000	854421	34221	888642	3.85	96.97
0.050	-1.30103	839183	32509	871692	3.73	93.91
0.50	-0.30103	715749	23844	739593	3.22	81.19
1.5	0.17609	790034	22804	812838	2.81	70.65
3.0	0.47712	873876	22586	896462	2.52	63.45
6.0	0.77815	761472	12265	773737	1.59	39.92
15	1.17609	863089	7750	870839	0.89	22.41
41	1.61278	857814	2266	860080	0.26	6.63
100	2.00000	826374	0	826374	0.00	0.00

No inhibitor run data used for normalization of enzyme activity

6'-SL Area	Lactose Area	Total Area	**Conversion (%)**
842842	34854	877696	3.97

[a]*Abbreviations: DANA 2,3-dehydro-2-deoxy-N-acetylneuraminic acid (DANA), 6'-sialyllactose (6'-SL). A 400 s push step and an additional 200 s 103 kPa (15 psi) 20% nanogel post plug were used after the enzyme zone was patterned to mitigate the effects of nanogel expansion.*

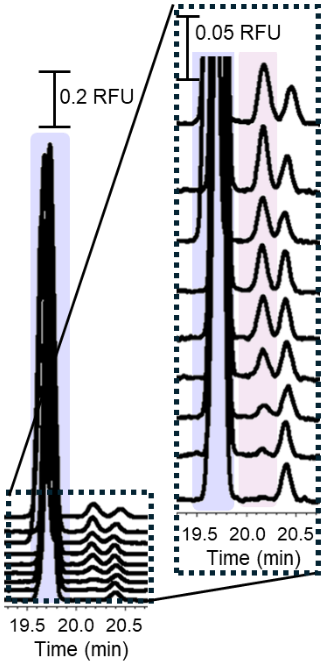

Figure S2A. DANA IC$_{50}$ curve 1 with concentrations ranging from 0.01 μM – 100 μM in descending order. Traces are offset in the x-axis and y-axis for visualization purposes. The x-axis offsets from top to bottom are 0, 0.95, -0.2, 0.55, 0.65, 0.61, -0.02, 0.38, and 0.18 minutes. The y-axis offsets from top to bottom are 0, -0.046, -0.075, -0.135, -0.165, -.195, -0.215, -0.245, -0.28 RFU.

Table S7B. DANA IC_{50} Curve #2 Results and Dose-response Inputs[a]

log[Inhibitor] (x-axis)		% Activity (y-axis input)				
[DANA] (µM)	**log[DANA] (µM)**	6'-SL Area	Lactose Area	Total Area	Conversion (%)	**Remaining Activity (%)**
0.010	-2.00000	758509	36529	795038	4.59	107.99
0.050	-1.30103	836752	36086	872838	4.13	97.17
0.50	-0.30103	598434	22401	620835	3.61	84.80
1.5	0.17609	775006	28968	803974	3.60	84.68
3.0	0.47712	709357	18084	727441	2.49	58.43
6.0	0.77815	774931	14458	789389	1.83	43.05
15	1.17609	718843	6380	725223	0.88	20.68
41	1.61278	693686	0	693686	0.00	0.00
100	2.00000	534015	0	534015	0.00	0.00

No inhibitor run data used for normalization of enzyme activity			
6'-SL Area	Lactose Area	Total Area	**Conversion (%)**
793939	35282	829221	4.25

[a]*Abbreviations: DANA 2,3-dehydro-2-deoxy-N-acetylneuraminic acid (DANA), 6'-sialyllactose (6'-SL). A 400 s push step and an additional 200 s 103 kPa (15 psi) 20% nanogel post plug were used after the enzyme zone was patterned to mitigate the effects of nanogel expansion.*

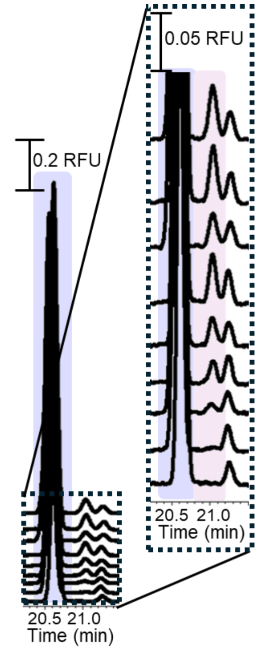

Figure S2B. DANA IC_{50} curve 2 with concentrations ranging from 0.01 µM – 100 µM in descending order. Traces are offset in the x-axis and y-axis for visualization purposes. The x-axis offsets from top to bottom are 0, 0.93, -0.63, 0.32, -0.015, 0.23, -0.42, 0.01, and -0.86 minutes. The y-axis offsets from top to bottom are 0, -0.06, -0.09, -0.16, -0.19, -0.22, -0.255, -0.29, and -0.31 RFU.

Table S7C. DANA IC_{50} Curve #3 Results and Dose-response Inputs[a]

log[Inhibitor] (x-axis)		% Activity (y-axis input)				
[DANA] (µM)	**log[DANA] (µM)**	6'-SL Area	Lactose Area	Total Area	Conversion (%)	**Remaining Activity (%)**
0.010	-2.00000	525049	6019	531068	1.13	91.94[b]
0.050	-1.30103	516697	6330	523027	1.21	98.17[b]
0.50	-0.30103	1085456	10411	1095867	0.95	77.06[b]
1.5	0.17609	1111258	7814	1119072	0.70	56.64[b]
3.0	0.47712	1149303	6261	1155564	0.54	43.95[b]
6.0	0.77815	851097	4677	855774	0.55	43.44[c]
15	1.17609	964421	3449	967870	0.36	28.91[b]
41	1.61278	977930	0	977930	0.00	0.00[b]
100	2.00000	950488	0	950488	0.00	0.00[b]

No inhibitor run data used for normalization of enzyme activity

6'-SL Area	Lactose Area	Total Area	**Conversion (%)**
1019977	12731	1032708	1.23[b]
902232	11495	913727	1.26[c]

[a]*Abbreviations: DANA 2,3-dehydro-2-deoxy-N-acetylneuraminic acid (DANA), 6'-sialyllactose (6'-SL)*
[b,c]*Indicates which no inhibitor run was used for the calculation of remaining activity*

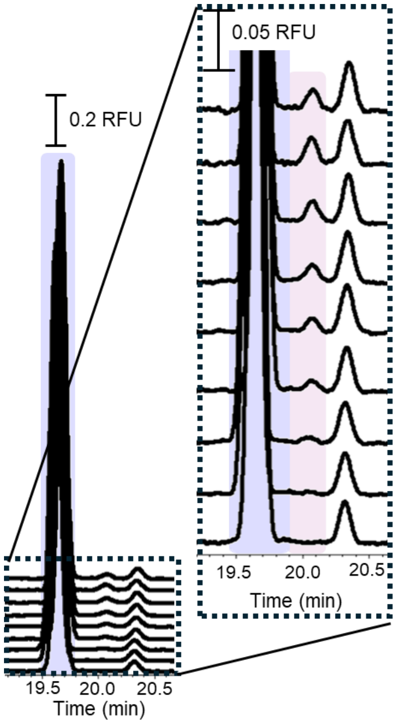

Figure S2C. DANA IC_{50} curve 3 with concentrations ranging from 0.01 µM – 100 µM in descending order. Traces are offset in the x-axis, y-axis, and when required scaled in the y-axis for visualization purposes. The x-axis offsets from top to bottom are 0, 0.19, 0.37, -0.09, 0.01, 0.15, -1, 0.15, -1, 0.03, and -0.03 minutes. The y-axis offsets from top to bottom are 0, -0.06, -0.1, -0.15, -0.2, -0.25, -0.3, -0.35, and -0.39 RFU. The y-axis scale offsets from top to bottom are 2, 2, 1, 1, 1, 1.2, 1, 1, and 1.

Table S7D. DANA IC_{50} Curve #4 Results and Dose-response Inputs[a]

log[Inhibitor] (x-axis)		% Activity (y-axis input)				
[DANA] (µM)	**log[DANA] (µM)**	6'-SL Area	Lactose Area	Total Area	Conversion (%)	**Remaining Activity (%)**
0.010	-2.00000	433343	6399	439742	1.46	101.98
0.050	-1.30103	406530	6442	412972	1.56	109.32
0.50	-0.30103	990705	11887	1002592	1.19	83.09
1.5	0.17609	955616	7677	963293	0.80	55.85
3.0	0.47712	507515	3883	511398	0.76	53.21
6.0	0.77815	816448	4829	821277	0.59	41.21
15	1.17609	850658	3191	853849	0.37	26.19
41	1.61278	917290	0	917290	0.00	0.00
100	2.00000	853278	0	853278	0.00	0.00

No inhibitor run data used for normalization of enzyme activity

6'-SL Area	Lactose Area	Total Area	**Conversion (%)**
899952	13027	912979	1.43

[a] *Abbreviations: DANA 2,3-dehydro-2-deoxy-N-acetylneuraminic acid (DANA), 6'-sialyllactose (6'-SL)*

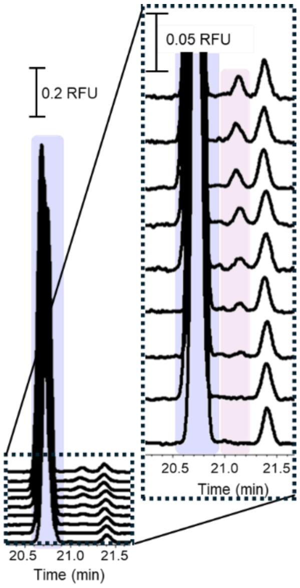

Figure S2D. DANA IC_{50} curve 4 with concentrations ranging from 0.01 µM – 100 µM in descending order. Traces are offset in the x-axis, y-axis, and when required scaled in the y-axis for visualization purposes. The x-axis offsets from top to bottom are 0, 0.34, 0.47,-0.16, -0.037, 0.74, 1, 0.16, and 0.29 minutes. The y-axis offsets from top to bottom are 0, -0.048,-0.078, -0.11, -0.16, -0.2, -0.24, -0.28, -0.32 RFU. The y-axis scale offsets from top to bottom are 1.8, 1.8, 1, 1, 1.8, 1, 1, 1, and 1.

Table S7E. DANA IC_{50} Curve #5 Results and Dose-response Inputs[a]

log[Inhibitor] (x-axis)		% Activity (y-axis input)				
[DANA] (µM)	**log[DANA] (µM)**	6'-SL Area	Lactose Area	Total Area	Conversion (%)	**Remaining Activity (%)**
0.010	-2.00000	70305	17118	720171	2.38	93.39
0.050	-1.30103	620241	16139	636380	2.54	99.64
0.50	-0.30103	1369969	28059	1398028	2.01	78.86
1.5	0.17609	1280930	21850	1302780	1.68	65.90
3.0	0.47712	1201621	15532	1217153	1.28	50.14
6.0	0.77815	1065833	11498	1077331	1.07	41.93
15	1.17609	1023128	5055	1028183	0.49	19.32
41	1.61278	1089607	0	1089607	0.00	0.00
100	2.00000	1012919	0	1012919	0.00	0.00

No inhibitor run data used for normalization of enzyme activity

6'-SL Area	Lactose Area	Total Area	**Conversion (%)**
831564	21717	853281	2.55

[a]*Abbreviations: DANA 2,3-dehydro-2-deoxy-N-acetylneuraminic acid (DANA), 6'-sialyllactose (6'-SL)*

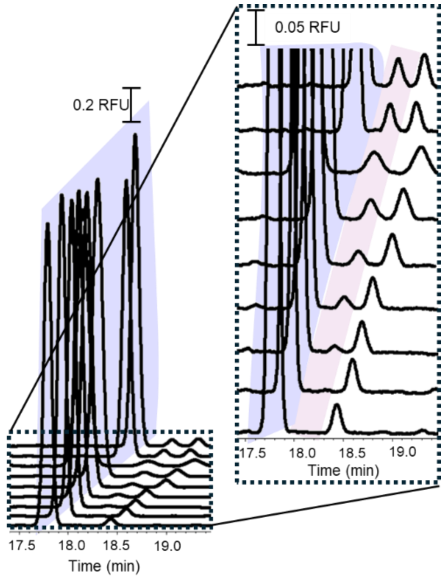

Figure S2E. DANA IC_{50} curve 5 with concentrations ranging from 0.01 µM – 100 µM in descending order. Traces are offset in the x- axis, y-axis, and when required scaled in the y axis for visualization purposes. The x-axis offsets from top to bottom are 0, -0.19, -3.4, -1.9, -2.15, -1.04, -0.7, -0.83, and -1.1 minutes. The y-axis offsets from top to bottom are 0.48, 0.42, 0.37, 0.34, 0.25, 0.18, 0.12, 0.07, and 0.02 RFU. The y- scale offsets from top to bottom are 1.5, 1.5, 1, 1, 1, 1, 1, 1, and 1.

Table S8A. Oseltamivir Acid IC_{50} Curve #1 Results and Dose-response Inputs

log[Inhibitor] (x-axis)		% Activity (y-axis input)				
[Oseltamivir Acid] (nM)	**log[Oseltamivir Acid] (nM)**	6'-SL Area[a]	Lactose Area	Total Area	Conversion (%)	**Remaining Activity (%)**
0.01	-2.00000	487211	7081	494292	1.43	104.55[b]
0.1	-1.00000	742460	9678	752138	1.29	93.91[b]
5	0.69897	1091174	13903	1105077	1.26	84.26[c]
13	1.11394	868583	7811	876394	0.89	59.69[c]
28	1.44716	888032	6191	894223	0.69	46.37[c]
33	1.51851	827075	2486	829561	0.30	20.07[c]
300	2.47712	705804	0	705804	0.00	0.00[c]
1000	3.00000	1000065	0	1000065	0.00	0.00[c]

No inhibitor run data used for normalization of enzyme activity

6'-SL Area	Lactose Area	Total Area	**Conversion (%)**
1236570	17179	1253749	1.37[b]
1170351	17739	1188090	1.49[c]

[a]Abbreviation: 6'-sialyllactose (6'-SL)
[b,c]Indicates which no inhibitor run was used for the calculation of remaining activity

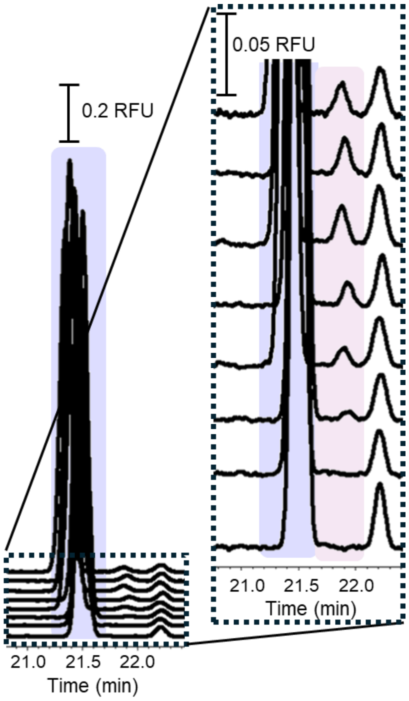

Figure S3A. Oseltamivir acid IC_{50} curve 1 with concentrations ranging from 0.01 nM – 1 μM in descending order. Traces are offset in the x-axis, y-axis, and when required scaled in the y axis for visualization purposes. The x- axis offsets from top to bottom are 0, 0, 0.66, 0.63, 0.71, 1.78, 1.6, and 1.78 minutes. The y-axis offsets from top to bottom are 0, -0.2, -0.048, -0.085, -0.115, -0.15, -0.18, and -0.22 RFU. The y-scale offset from top to bottom are 1.9, 1.4, 1,1,1,1,1,and 1.

Table S8B. Oseltamivir Acid IC_{50} Curve #2 Results and Dose-response Inputs

log[Inhibitor] (x-axis)		% Activity (y-axis input)				
[Oseltamivir Acid] (nM)	**log[Oseltamivir Acid] (nM)**	6'-SL Area[a]	Lactose Area	Total Area	Conversion (%)	**Remaining Activity (%)**
0.01	-2.00000	929861	14563	944424	1.54	95.22[b]
0.1	-1.00000	1606800	25064	1631964	1.54	94.84[b]
5	0.69897	1445669	24275	1469944	1.65	82.66[c]
13	1.11394	1376172	18007	1394179	1.29	64.65[c]
28	1.44716	1499760	11810	1511570	0.78	39.11[c]
33	1.51851	1326914	7769	1334683	0.58	29.14[c]
300	2.47712	1505420	0	1505420	0.00	0.00[c]
1000	3.00000	1524018	0	1524018	0.00	0.00[c]

No inhibitor run data used for normalization of enzyme activity

6'-SL Area	Lactose Area	Total Area	**Conversion (%)**
1300543	21408	1321951	1.62[b]
1380985	28151	1409136	2.00[c]

[a]*Abbreviation: 6'-sialyllactose (6'-SL)*
[b,c]*Indicates which no inhibitor run was used for the calculation of remaining activity*

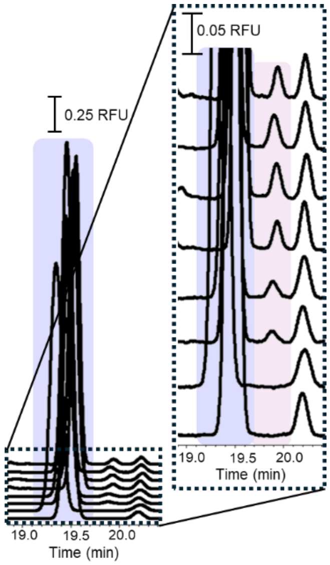

Figure S3B. Oseltamivir acid IC_{50} curve 2 with concentrations ranging from 0.01 nM – 1 μM in descending order. Traces are offset in the x-axis, y-axis, and when required scaled in the y-axis for visualization purposes. The x-axis offsets from top to bottom are 0, -0.62, -1.35, 0.08, -1.3, -1.1, -1.65, -0.58 minutes. The y-axis offsets from top to bottom are 0, -0.055, -0.11, -0.17, -0.225, -0.275, -0.33, and -0.38 RFU. The y-scale offsets from top to bottom are 1.3, 1, 1, 1, 1, 1, 1, and 1.

Table S8C. Oseltamivir Acid IC_{50} Curve #3 Results and Dose-response Inputs						
log[Inhibitor] (x-axis)		% Activity (y-axis input)				
[Oseltamivir Acid] (nM)	**log[Oseltamivir Acid] (nM)**	6'-SL Area[a]	Lactose Area	Total Area	Conversion (%)	**Remaining Activity (%)**
0.01	-2.00000	691763	13055	704818	1.85	102.81[b]
0.1	-1.00000	1907915	38264	1946179	1.97	109.13[b]
5	0.69897	1488575	23251	1511826	1.54	85.37[b]
13	1.11394	1632640	21378	1654018	1.29	63.18[c]
28	1.44716	530307	3930	534237	0.74	35.96[c]
33	1.51851	647481	3464	650945	0.53	26.01[c]
300	2.47712	520053	0	520053	0.00	0.00[c]
1000	3.00000	1340274	0	1340274	0.00	0.00[c]

No inhibitor run data used for normalization of enzyme activity			
6'-SL Area	Lactose Area	Total Area	**Conversion (%)**
1396058	25612	1421670	1.80[b]
1287453	26889	1314342	2.05[c]

[a]Abbreviation: 6'-sialyllactose (6'-SL)
[b,c]Indicates which no inhibitor run was used for the calculation of remaining activity

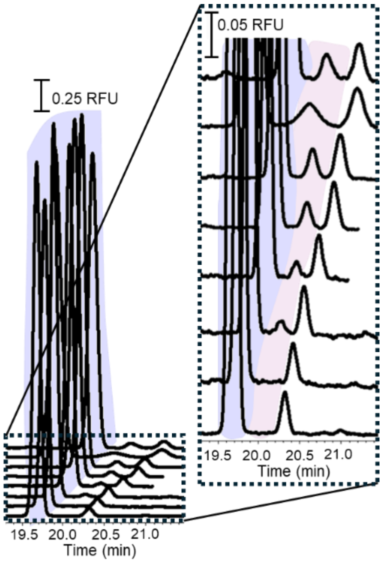

Figure S3C. Oseltamivir acid IC_{50} curve 3 with concentrations ranging from 0.01 nM – 1 µM in descending order. Traces are offset in the x-axis, y-axis, and when required scaled in the y-axis for visualization purposes. The x-axis offsets from top to bottom are 0, -0.9, -0.005, -0.04, 0.75, 0.45, 0.4, and 0.45 minutes. The y-axis offsets from top to bottom are 0, -0.06, -0.125, -0.19, -0.255, -0.32, -0.37, and -0.44 RFU. The y-scale offsets from top to bottom are 2, 1, 1, 1, 2.5, 2.2, 2.3, and 1.

Table S9A. Peramivir IC_{50} Curve #1 Results and Dose-response Inputs

log[Inhibitor] (x-axis)		% Activity (y-axis input)				
[Peramivir] (nM)	**log[Peramivir] (nM)**	6'-SL Area[a]	Lactose Area	Total Area	Conversion (%)	**Remaining Activity (%)**
2.7	0.43136	1005881	41023	1046904	3.92	99.62[b]
5.4	0.73239	1091772	48705	1140477	4.27	108.57[b]
16	1.20412	843947	26423	870370	3.04	77.96[c]
68	1.83251	1170840	28208	1199048	2.35	60.41[c]
95	1.97772	1052594	20620	1073214	1.92	41.11[d]
161	2.20683	941745	10724	952469	1.13	24.09[d]
543	2.73480	984617	0	984617	0.00	0.00[c]
1086	3.03583	1145940	0	1145940	0.00	0.00[c]

No inhibitor run data used for normalization of enzyme activity			
6'-SL Area	Lactose Area	Total Area	**Conversion (%)**
901040	36892	937932	3.93[b]
1181351	47868	1229219	3.89[c]
1048315	69044	1477359	4.67[d]

[a]Abbreviation: 6'-sialyllactose (6'-SL)
[b,c,d]Indicates which no inhibitor run was used for the calculation of remaining activity

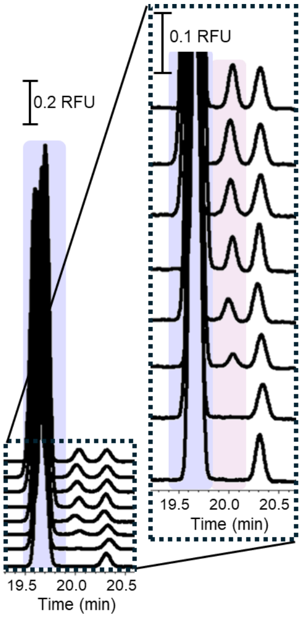

Figure S4A. Peramivir IC_{50} curve 1 with concentrations ranging from 3 nM – 1.09 µM in descending order. Traces are offset in the x-axis, y-axis, and when required scaled in the y-axis for visualization purposes. The x- axis offsets from top to bottom are 0, -0.58, 0.01, 0.8, 0.46, 0.31, 0.061, and 0.35 minutes. The y-axis offsets from top to bottom are 0, -0.09, -0.19, -0.27, -0.35, -0.44, -0.52, and -0.62 RFU. The y-scale offsets from top to bottom are 1, 1, 1.3, 1, 1, 1, 1, and 1.

Table S9B. Peramivir IC_{50} Curve #2 Results and Dose-response Inputs

log[Inhibitor] (x-axis)		% Activity (y-axis input)				
[Peramivir] (nM)	**log[Peramivir] (nM)**	6'-SL Area[a]	Lactose Area	Total Area	Conversion (%)	**Remaining Activity (%)**
2.7	0.43136	1344364	23283	1367647	1.70	97.90[b]
5.4	0.73239	1267577	25057	1292634	1.94	111.47[b]
16	1.20412	1286637	17826	1304463	1.37	74.31[c]
68	1.83251	1234172	13552	1247724	1.09	59.06[c]
95	1.97772	1105442	7289	1112731	0.66	31.64[d]
161	2.20683	1089132	5343	1094475	0.49	23.58[d]
543	2.73480	1092471	0	1092471	0.00	0.00[d]
1086	3.03583	1205348	0	1205348	0.00	0.00[d]

No inhibitor run data used for normalization of enzyme activity

6'-SL Area	Lactose Area	Total Area	**Conversion (%)**
1375840	24348	1400188	1.74[b]
1384238	25934	1410172	1.84[c]
1197756	25318	1223074	2.07[d]

[a]*Abbreviation: 6'-sialyllactose (6'-SL)*
[b,c,d]*Indicates which no inhibitor run was used for the calculation of remaining activity*

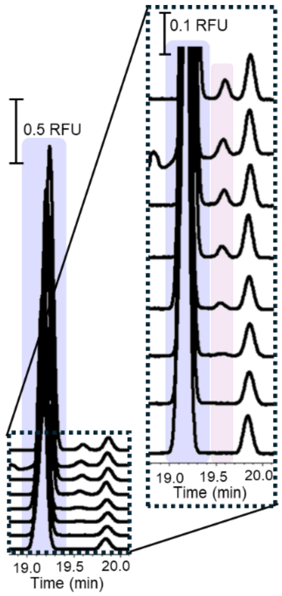

Figure S4B. Peramivir IC_{50} curve 2 with concentrations ranging from 3 nM – 1.09 μM in descending order. Traces are offset in the x-axis and y-axis for visualization purposes. The x-axis offsets from top to bottom are 0, 0.057, -0.014, 0.007, -0.3, -0.185,-0.4, and -0.46 minutes. The y-axis offsets from top to bottom are 0,-0.13, -0.23, -0.34, -0.455, -0.555, -0.67, and -0.77 RFU.

Table S9C. Peramivir IC_{50} Curve #3 Results and Dose-response Inputs

log[Inhibitor] (x-axis)		% Activity (y-axis input)				
[Peramivir] (nM)	**log[Peramivir] (nM)**	6'-SL Area[a]	Lactose Area	Total Area	Conversion (%)	**Remaining Activity (%)**
2.7	0.43136	1269676	20993	1290669	1.63	94.95[b]
5.4	0.73239	1304488	21632	1326120	1.63	95.22[b]
16	1.20412	847025	11043	858068	1.29	75.13[b]
68	1.83251	1366629	14140	1380769	1.02	55.98[c]
95	1.97772	1065234	7476	1072710	0.70	38.10[c]
161	2.20683	864618	2185	866803	0.25	13.78[c]
543	2.73480	779465	0	779465	0.00	0.00[c]
1086	3.03583	1197090	0	1197090	0.00	0.00[c]

No inhibitor run data used for normalization of enzyme activity

6'-SL Area	Lactose Area	Total Area	**Conversion (%)**
1311805	24445	1336250	1.83[b]
1302034	22693	1324727	1.71[c]

[a]*Abbreviation: 6'-sialyllactose (6'-SL)*
[b,c]*Indicates which no inhibitor run was used for the calculation of remaining activity*

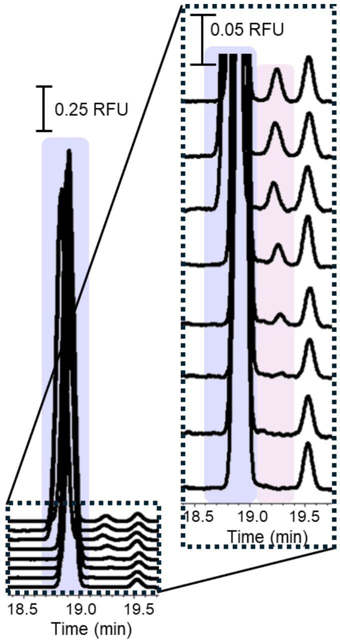

Figure S4C. Peramivir IC_{50} curve 3 with concentrations ranging from 3 nM – 1.09 µM in descending order. Traces are offset in the x-axis, y-axis, and when required scaled in the y-axis for visualization purposes. The x-axis offsets from top to bottom are 0, -0.63, -0.26, -0.38, -0.4, -0.26, -0.41, and -0.35 minutes. The y-axis offsets from top to bottom are 0, -0.061, -0.13, -0.174, -0.23, -0.3, -0.36, and -0.41 RFU. The y-scale offsets from top to bottom are 1, 1, 1.6, 1, 1, 1.2, 1.4, and 1.

Table S10A. Resolution Achieved with Increased Nanogel Concentration

	% Nanogel	6'-Siallylactose Time (min)	6'-Siallylactose WHM[a] (min)	Lactose Time (min)	Lactose WHM[a] (min)	Resolution[b]
Set 1	5	13.800	0.144	13.979	-[c]	-[c]
	10	14.733	0.130	14.996	0.138	1.16
	15	15.825	0.121	16.192	0.125	1.76
	20	17.600	0.119	18.129	0.121	2.60
	25	20.808	0.158	21.738	0.183	3.22
Set 2	5	14.104	0.142	14.371	-[c]	-[c]
	10	15.217	0.128	15.492	0.133	1.24
	15	16.300	0.122	16.679	0.130	1.77
	20	18.125	0.126	18.688	0.128	2.62
	25	20.967	0.158	21.817	0.159	3.16
Set 3	5	14.200	0.140	14.521	-[c]	-[c]
	10	15.300	0.124	15.575	0.134	1.26
	15	16.529	0.115	16.917	0.125	1.91
	20	18.450	0.121	19.012	0.126	2.68
	25	22.387	0.168	23.321	0.169	3.27
Set 4	5	14.375	0.139	14.750	-[c]	-[c]
	10	15.350	0.123	15.625	0.130	1.28
	15	16.637	0.114	17.025	0.132	1.86
	20	18.667	0.121	19.229	0.118	2.77
	25	22.392	0.155	23.363	0.188	3.34

[a] *Width at half maximum height (WHM)*
[b] *Resolution is calculated as 1.18*(time lactose- time sialyllactose)/(WHM lactose + WHM sialyllactose).*
[c] *The WHM cannot be determined using automatic peak detection with the data processing software.*

Table S10B. Summary of resolution data in Table S10A

% Nanogel	Resolution (n = 4)
**10**	$1.23_5 \pm 0.05_4$ (4% RSD)
**15**	$1.82_6 \pm 0.07_0$ (4% RSD)
**20**	$2.66_9 \pm 0.07_9$ (3% RSD)
**25**	$3.24_8 \pm 0.07_5$ (2% RSD)

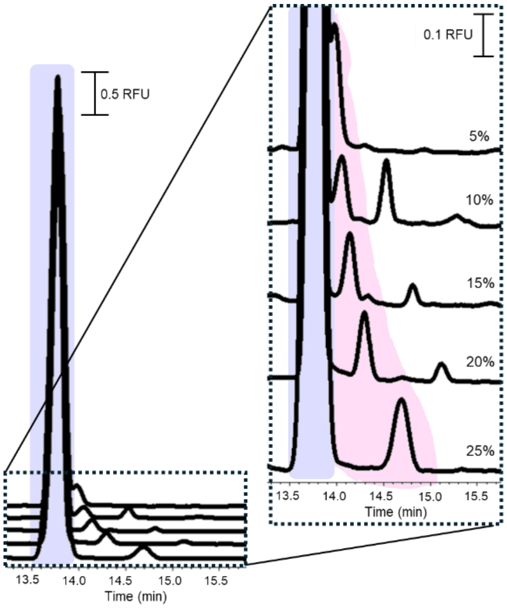

Figure S5. Altering the percent nanogel to achieve a resolution above 3.0. Traces are offset in the x-axis and y-axis for the purpose of visualization. From top to bottom percentages read 5, 10, 15, 20, 25%. Offsets in the x-axis from top to bottom are 0, -0.94, -2.05, -3.83, -7.045. Offsets in the y-axis from top to bottom are 0, -0.15, -0.31, -0.465, -0.65.

Table S11A. Effect of 0 mM NaCl on H5N1 Neuraminidase Conversion[a]								
[Per-amivir] nM	6'-SL Area (SL)	Lactose Area (L_p)	Lactose Cont. (L_c)	Total Area (L_p+L_c)	Total Area ($SL+L_p+L_c$)	Conversion (%) (L_p+L_c)	Conversion to Lactose (%) (L_p)[b]	Activity Remain (%)[c]
Blank	4375159	0	91083	91083	4466242	2.04	*NA*	*NA*
0 nM	2973382	359593	59349	418942	3392324	10.60	8.56[d]	100
	3748684	380483	81050	461533	4210217	9.04	7.00[e]	100
	4054543	424928	85774	510702	4565245	9.31	7.27[f]	100
	Average L_p Conversion: *7.6₀ ± 0.8₃ (10% RSD)*[g]							
1 nM	3601295	231372	84317	315689	3916984	5.91	3.87[d]	45.18[d]
	3976748	283406	88036	371442	4348190	6.52	4.48[e]	64.00[e]
	4207257	290917	92828	383745	4591002	6.34	4.30[f]	59.12[f]
	Average Activity Remaining: *56.0 ± 9.7 (20% RSD)*[h]							

[a] *Abbreviation: 6'-sialyllactose (6'-SL), 6'-sialyllactose substrate (SL), lactose product (L_p), lactose contaminant present in sample prior to enzyme reaction (L_c).*

[b] *The calculation of the percent enzyme conversion (i.e. Conversion to Lactose, L_p) is modified for this study because the peaks for enzymatically converted lactose and lactose contaminant in the sample are not baseline resolved. For this calculation, the percent contribution of the contaminant peak area (L_c) is measured in the absence of enzyme (i.e. Blank) and calculated as the L_c area divided by the total as follows (Area-L_c)/(Area-SL+ Area-L_c). This value is the amount of lactose contaminant (% area L_c) that is subtracted from the amount of lactose product and lactose contaminant (% area of L_p+L_c) which is the measured as the unresolved peak areas of lactose and contaminant observed following the enzyme reaction. The calculation of the this corrected percent conversion (Area-L_p)/(Area-SL+Area-L_p) is as follows: (Area-L_p)/(Area-SL+Area-L_p) =((L_p+L_c)/($SL+L_p+L_c$))-((L_c)/($SL+L_c$)).*

[c,d,e,f] *The percent activity remaining is calculated as the percent conversion with inhibitor divided by the percent conversion without inhibitor. Indicates which no inhibitor run was used for the calculation of activity remaining.*

[g] *The averages of percent conversion for 0 mM NaCl (Table S11A) and 100 mM NaCl (Table 11SB) are statistically different (student's t-test, n = 3, 95 % confidence level).*

[h] *The averages of percent activity remaining for 0 mM NaCl (Table S11A) and 100 mM NaCl (Table 11SB) are statistically the same (student's t-test, n = 3, 95 % confidence level).*

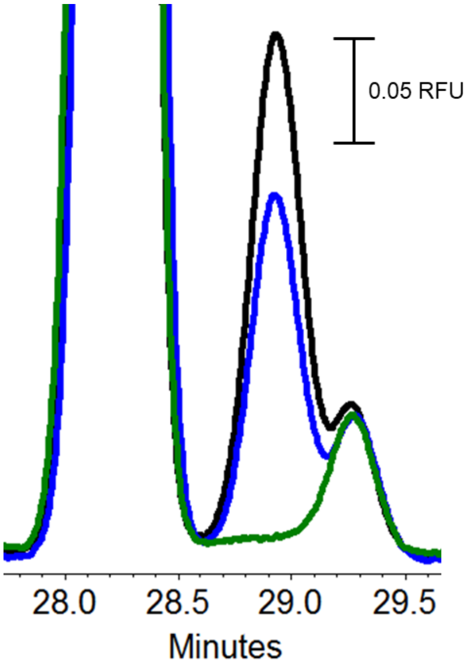

Figure S6A. Summary of the effect of 0 mM sodium chloride on enzyme activity and inhibition for neuraminidase from H5N1. The separations are performed in the absence (black) and presence (blue traces) of peramivir in a 25 µm inner diameter capillary. No enzyme (green) was used to subtract the contaminant for quantification of activity remaining. The blue and green traces were offset for the purpose of visualization. Blue was offset in the x-axis and y-axis by -0.185 and -0.0078 respectively. The green trace was offset in the x-axis and y-axis by 0.58 and -0.0068, respectively. The H5N1 salt study of separations with the background electrolyte (BGE) containing either 0 mM or 100 mM NaCl required a 25 µm inner diameter capillary for baseline resolution of the product peak under both conditions. Patterning was done at 15 °C. The capillary was flushed for 6 min at 172 kPa (25 psi) with the BGE composed of 50 mM MES, 5 mM $CaCl_2$ at pH 6.5, and then for 10 min 172 kPa (25 psi) with 30% nanogel fill. Next a 5% nanogel zone was introduced at 48 kPa (7 psi) for 41.8 s followed by an enzyme zone for 4.0 s at 34 kPa (5 psi). The 0.1 cm enzyme zone was then pushed 12 cm with 30% nanogel for 282.8 s at 48 kPa (7 psi). Next mixing in the capillary was done by pushing BGE at 48 kPa (7 psi) for 47.1 s in the reverse direction and then pushing 30% nanogel at 48 kPa (7 psi) for 47.1 s in the forward direction. Once this patterning was complete, the sample was electrokinetically injected (-8kV, 4s), followed by the injection of a post plug of 30% nanogel for 23.6 s with 48 kPa (7 psi) to prevent the analyte from being ejected from the separation capillary. The capillary temperature was raised to 20 °C during a 3-minute wait step. The substrate was driven towards the enzyme zone with an applied voltage of -10 kV for 1.3 min. The capillary temperature was then raised to 37 °C during a 4-minute wait step and then electrokinetically driven through the enzyme zone at -8kV for 9.9 minutes. Finally, the capillary temperature was switched to 20 °C during a 4-minute wait step and then the substrate and product were electrokinetically driven to the detection window at -10 kV.

Table S11B. Effect of 100 mM NaCl on H5N1 Neuraminidase

[Peramivir] nM	6'-Sialyllactose Area	Lactose Area	Total Area	Conversion (%)[a]	Activity Remaining (%)[b]
No Inhibitor	10391417	74902	10466319	0.72[c]	100
	9094367	52915	9147282	0.58[d]	100
	9535653	44403	9580056	0.46[e]	100
Average Conversion: *$0.5_8 \pm 0.1_2$ (20% RSD)*[f]					
1 nM	9929082	52382	9981464	0.52[c]	73.33[c]
	8471695	32307	8504002	0.38[d]	65.67[d]
	10352801	33483	10386284	0.32[e]	69.55[e]
Average Activity Remaining: *$69._5 \pm 3._8$ (6% RSD)*[g]					

[a]*The percent enzyme conversion is calculated as the area of lactose divided by total area.*
[b]*The percent activity remaining is calculated as the percent conversion with inhibitor divided by the percent conversion without inhibitor.*
[c,d,e]*Indicates which no inhibitor run was used for the calculation of activity remaining.*
[f]*The averages of percent conversion for 0 mM and 100 NaCl (Table S11A,B) are statistically different (student's t-test, n = 3, ρ = 0.05).*
[g]*The averages of percent activity remaining for 0 mM and 100 NaCl (Table S11A,B) are statistically the same (student's t-test, n = 3, ρ = 0.05).*

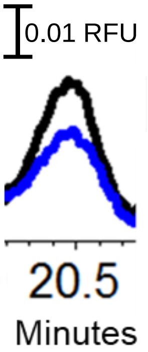

Figure S6B. Summary of the effect of 100 mM sodium chloride on enzyme activity and inhibition for neuraminidase from H5N1. The separations are performed in the absence (black) and presence (blue traces) of Peramivir in a 25 µm inner diameter capillary. The blue trace was offset for the purpose of visualization in the x-axis and y-axis by 0.25 and 0.002, respectively. The H5N1 salt study of separations with the background electrolyte (BGE) containing either 0 mM or 100 mM NaCl required a 25 µm inner diameter capillary for baseline resolution of the product peak under both conditions. Patterning was done at 15 °C. The capillary was flushed for 6 min at 172 kPa (25 psi) with the BGE composed of 50 mM MES, 5 mM $CaCl_2$, 100 mM NaCl at pH 6.5, and then for 10 min 172 kPa (25 psi) with 30% nanogel fill. Next a 5% nanogel zone was introduced at 48 kPa (7 psi) for 41.8 s followed by an enzyme zone for 4.0 s at 34 kPa (5 psi). The 0.1 cm enzyme zone was then pushed 12 cm with 30% nanogel for 282.8 s at 48 kPa (7 psi). Next mixing in the capillary was done by pushing BGE at 48 kPa (7 psi) for 47.1 s in the reverse direction and then pushing 30% nanogel at 48 kPa (7 psi) for 47.1 s in the forward direction. Once this patterning was complete, the sample was electrokinetically injected (-8kV, 4s), followed by the injection of a post plug of 30% nanogel for 23.6 s with 48 kPa (7 psi) to prevent the analyte from being ejected from the separation capillary. The capillary temperature was raised to 20 °C during a 3-minute wait step. The substrate was driven towards the enzyme zone with an applied voltage of -10 kV for 6.8 min. The capillary temperature was then raised to 37 °C during a 4-minute wait step and then electrokinetically driven through the enzyme zone at -2kV for 30 minutes. Using the lower applied voltage during the step to drive the substrate through the enzyme zone increased the conversion as has been previously reported (Gattu, S.; Crihfield, C. L.; Holland, L. A., Analytical Chemistry 2017, 89(1), 929-936.). Finally, the capillary temperature was switched to 20 °C during a 4-minute wait step and then the substrate and product were electrokinetically driven to the detection window at -10 kV.

Table S12 Zone Study of Peramivir and H5N1					
	6'-SL Area[a]	Lactose Area	Total Area	% Conversion[b]	% Activity Remaining[c]
**Set 1 Patterning with 0 nM Peramivir Inhibitor in the Enzyme Stock (Zone 2)**					
No Inhibitor 1	6882183	559134	7441317	7.51	100.00
Inhibitor in zones 1, 3, and 4	7798460	353419	8151879	4.34	57.70
	7694725	317538	8012263	3.96	52.74
	7841602	321466	8163068	3.94	52.41
Average Activity Remaining: 54.3 ± 2.9 (5% RSD)[d]					
**Set 2 Patterning with 5.4 nM Peramivir Inhibitor in the Enzyme Stock (Zone 2)**					
No Inhibitor 2	8029965	423786	8453751	5.01	100.00
Inhibitor in zones 1, 2, 3, and 4	9478088	208191	9686279	2.15	42.88
	9354311	323222	9677533	3.34	66.63
	6308774	227408	6536182	3.48	69.40
Average Activity Remaining: 60 ± 10 (20% RSD)[d]					

[a] Abbreviations: 6'-SL 6'-sialyllactose

[b] The percent enzyme conversion is calculated as the area of lactose divided by total area.

[c] The percent activity remaining for set 1 vs. set 2 is calculated as the percent conversion with inhibitor divided by the percent conversion without inhibitor. The runs obtained in the absence of inhibitor for set 1 (No Inhibitor 1) and for set 2 (No Inhibitor 2) were done immediately before the set of replicate runs and used to normalize the percent conversion for each set.

[d] The average percent activity remaining with inhibitor in zones 1, 3, and 4 is statistically the same as that obtained with inhibitor in zones 1, 2, 3, and 4 (student's t-test, n = 3, 95 % confidence level).

Table S13. Peramivir H_5N_1 IC_{50} Curve Results and Dose-response Inputs

log[Inhibitor] (x-axis)		% Activity (y-axis input)				
[Peramivir] (nM)	**log[Peramivir] (nM)**	6'-SL Area[a]	Lactose Area	Total Area	Conversion (%)	**Remaining Activity (%)**
**0.01**	-2.00000	3129762	62673	3192435	1.96	82.52
**1.0**	0.00000	2428825	45323	2474148	1.83	77.00
**3.0**	0.47712	2154798	36908	2191706	1.68	70.78
**5.0**	0.69897	2639743	40091	2679834	1.50	62.88
**10.0**	1.00000	3380425	24769	3405194	0.73	30.57
**13.5**	1.13033	4432707	13418	4446125	0.30	12.68
**75**	1.87506	3016631	0.00	3016631	0.00	0.00
**750**	2.87506	3231570	0.00	3231570	0.00	0.00

No inhibitor run data used for normalization of enzyme activity			
6'-SL Area	Lactose Area	Total Area	**Conversion (%)**
2429815	59217	2489032	2.38

[a]*Abbreviations: 6'-sialyllactose (6'-SL)*

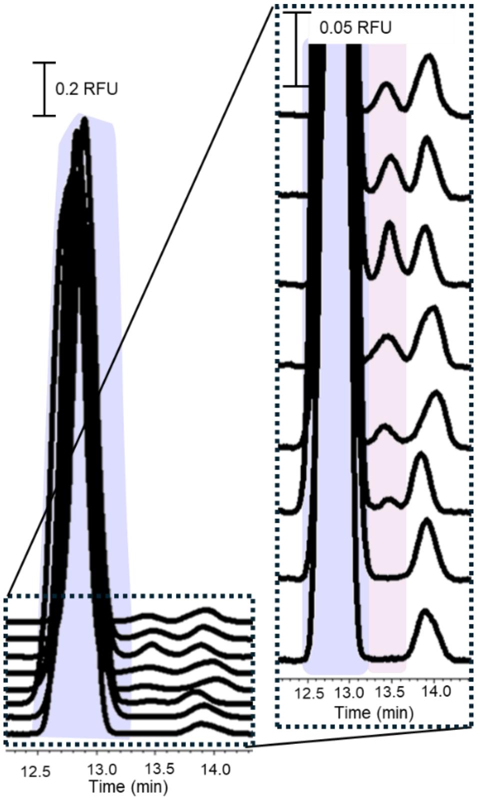

Figure S7. H_5N_1 Peramivir IC_{50} curve with concentrations ranging from 0.01 nM - 750 nM in descending order. Traces are offset in the x-axis and y-axis for the purpose of visualization. The x-axis offsets from top to bottom are 0.04, 1.5, 0.15, -0.017, 0, 0.02, 0.37, and -0.36 minutes. The y-axis offsets from top to bottom are -0.08, -0.01, 0.05, -0.14, -0.2, -0.25, -0.3, and -0.36 RFU. Patterning was done at 15 °C. The capillary was flushed for 5 min at 689 kPa (100 psi) with the background electrolyte (BGE) composed of 50 mM MES, 100 mM NaCl, and 5 mM CaCl2 at pH 6.5, and then for a 20 min 517 kPa (75 psi) with 25% nanogel. Next a 5 % nanogel zone was introduced at 103 kPa (15 psi) for 20 s followed by an enzyme zone for 6.3 s at 34 kPa (5 psi). The enzyme zone was then pushed 13 cm with 25% nanogel using 103 kPa (15 psi) for 830 s. Next mixing in the capillary was done by pushing BGE at 517 kPa (75 psi) for 14.8 s in the reverse direction and then pushing 25% nanogel at 517 kPa (75 psi) for 14.8 s in the forward direction. Once this patterning was complete, the sample was electrokinetically injected (-8kV, 4s), followed by the injection of a post plug of 25% nanogel for 35 s with 103 kPa (15 psi) to prevent the analyte from being ejected from the separation capillary. The capillary temperature was raised to 37 °C during a 5-minute wait step. The sample was electrokinetically driven through the capillary for 4 min at -12kV, then at -3kV for 18.9 minutes and finally at -12kV for the separation.
